# Investigation of Anticancer Properties of Newly Synthesized Pyridazine-Based Inhibitors in Mouse and Human Breast Cancer Cell Line

**DOI:** 10.3390/biology14091193

**Published:** 2025-09-04

**Authors:** Kübra Acikalin Coskun, Elif Cansu Abay, Mehmet Gumus, Ayşe Büşranur Çelik, Levent Gulum, Irfan Koca, Yusuf Tutar

**Affiliations:** 1 Division of Medicinal Biology, Department of Basic Medical Sciences, Istanbul Aydın University, 34295 Istanbul, Turkey; kubraacikalincoskun@aydin.edu.tr; 2 Division of Medical Biology, Hamidiye Health Institute, University of Health Sciences, 34668 Istanbul, Turkey; abycansu@gmail.com; 3 Department of Occupational Health and Safety, Akdağmadeni School of Health, 66900 Yozgat, Turkey; mehmet.gumus@yobu.edu.tr; 4 Division of Molecular Biology and Genetics, Hamidiye Health Institute, University of Health Sciences, 34668 Istanbul, Turkey; ayseclk1899@gmail.com; 5 Mudurnu Sureyya Astarcı Vocational School, Abant Izzet Baysal University, 14800 Bolu, Turkey; leventgulum@ibu.edu.tr; 6 Division of Organic Chemistry, Department of Chemistry, Faculty of Science, Yozgat Bozok University, 66100 Yozgat, Turkey; irfan.koca@yobu.edu.tr; 7 Division of Medicinal Biochemistry, Department of Basic Medical Sciences, Faculty of Medicine, Recep Tayyip Erdogan University, 53100 Rize, Turkey; 8 Division of Biochemistry, Department of Basic Pharmaceutical Sciences, Faculty of Pharmacy, University of Health Sciences, 34668 Istanbul, Turkey

**Keywords:** breast cancer, chemotherapy, Hsp90, DOX, inhibitor, anticancer effect

## Abstract

Background: Breast cancer is the most common cancer among women. Although doxorubicin (DOX) is widely used in its treatment, its dose-dependent toxicity and the development of drug resistance reduce its therapeutic efficacy. Therefore, this study aims to identify a novel anticancer agent that is more effective than DOX, inhibits cancer cell growth, and is less toxic to healthy cells. Methods: The cytotoxic effects of DOX and 2S-series molecules were evaluated on human (MDA-MB-231) and mouse (4T1) TNBC breast cancer cell lines and healthy breast epithelial (hTERT) cells using MTT assays at 48 and 72 h to screen functional similarities and possible differences upon drug/inhibitor treatment. Apoptosis and cell cycle analysis were analyzed by flow cytometry. Gene expression profiles were assessed by qPCR, and binding interactions with Hsp90 were examined via molecular docking. Results: 2S-5 exhibited IC_50_ values of 6.21 µM (MDA-MB-231) and 7.04 µM (4T1), while 2S-13 showed IC_50_ values of 7.73 µM and 8.21 µM, respectively. Both compounds demonstrated selective cytotoxicity against cancer cells. Gene expression and pathway analysis revealed that 2S-13 modulated the PI3K-Akt, MAPK, apoptosis, and HIF-1 pathways, showing broader modulation than DOX. Conclusions: 2S-13 appears to be a promising drug candidate, particularly in the MDA-MB-231 cell line. However, the current findings are limited to in vitro models. Further in vivo studies and pharmacokinetic analyses are required to validate its therapeutic potential, assess long-term efficacy and safety, and explore its resistance profile and molecular mechanisms in more detail.

## Simple Summary

Breast cancer, especially triple-negative breast cancer (TNBC), is one of the most aggressive cancer types and is difficult to treat effectively due to drug resistance and toxicity. Although doxorubicin (DOX) is commonly used in cancer treatment, it causes serious side effects on healthy tissues. This study investigates new drug candidates synthesized from aryl hydrazonal compounds that target the Hsp90 protein, a key molecule for cancer cell survival. Among the 2S-series compounds tested, 2S-13 showed strong anticancer effects, especially in the human TNBC cell line MDA-MB-231. Compared to DOX, 2S-13 had lower toxicity on healthy cells and higher selectivity toward cancer cells. Molecular experiments revealed that 2S-13 affects crucial cancer-related pathways such as PI3K/Akt, MAPK, and apoptosis. Flow cytometry results showed that 2S-13 causes cell cycle arrest in the G0/G1 phase and promotes apoptosis in cancer cells. Further, molecular docking results confirmed that 2S-13 binds more strongly to the ATP site of Hsp90 than that of DOX, suggesting a better inhibitory effect. In conclusion, 2S-13 is a promising anticancer candidate with high efficacy and low toxicity, especially for the treatment of triple-negative breast cancer.

## 1. Introduction

Triple-negative breast cancer (TNBC) accounts for approximately 15% of breast cancers and is defined by a loss of estrogen and progesterone receptor expression and the absence of HER2 amplification/overexpression. Due to the limited availability of early prognostic tools, frequent recurrence and metastatic spread, and the absence of validated targeted therapies, outcomes are poorer compared to those of other subtypes Despite the well-known cardiotoxicity and resistance in approximately 30–50% of patients, anthracyclines (e.g., doxorubicin) remain the standard of care [1,2,3].

Heat shock protein 90 (Hsp90) is an ATP-dependent molecular chaperone responsible for the folding, stabilization, and activation of numerous oncogenic client proteins, including key nodes in the PI3K–AKT and MAPK signaling pathways central to TNBC biology [4,5]. Pharmacological inhibition of the N-terminal ATP-binding pocket halts the Hsp90 chaperone cycle [5]; however, first-generation N-terminal Hsp90 inhibitors have shown limited clinical efficacy in breast cancer and frequently trigger an HSFthera1-mediated heat shock response (HSR), which upregulates Hsp70 and other chaperones, thereby weakening antitumor activity and contributing to toxicity [6,7]. Additionally, increased Hsp90 expression has been associated with adverse clinical–pathological features and poor prognosis in TNBC [4]. These observations support the rational design of new-generation Hsp90 inhibitors that can limit HSR induction and enhance target selectivity.

Pyridazine is a six-membered heterocyclic molecule with nitrogen atoms at positions 1 and 2. These nitrogen atoms give off chemical characteristics like chelating, hydrogen bonding, and protonation. These characteristics make pyridazine derivatives one of the structures that chemical and pharmaceutical research is most interested in [8]. Numerous studies in the literature demonstrate that appropriately functionalized pyridazine derivatives exhibit a wide range of systematic pharmacological effects, including analgesic [9], anti-inflammatory [10], antibacterial [11], antithrombotic [12], diuretic [13], antihypertensive [14], antidiabetic [15], and therapeutic for Alzheimer’s disease [16]. This ability is being considered a valuable structural resource for anticancer drug development through therapies that can target different stages of cancer [17,18].

The aim of this study was to identify a new anticancer agent that is more effective than doxorubicin (DOX) in inhibiting TNBC cell proliferation while exhibiting minimal toxicity against non-malignant hTERT cells. To this end, we designed and synthesized a 24-member library of pyridazine-centered aryl-hydrazone derivatives, tested their inhibitory effects on human MDA-MB-231 and mouse 4T1 TNBC cell lines using standard dose–response assays, and evaluated their selectivity against hTERT. In parallel, since Hsp90 keeps oncogenic proteins in an active state and supports survival signaling in cancer cells, we evaluated compound binding to the Hsp90 N-terminal ATP pocket in silico. Additionally, we elucidated the underlying mechanisms by examining the effects of the compounds and DOX on cancer pathways, cell cycle arrest, and apoptosis, and identified candidates with superior activity compared to DOX.

## 2. Materials and Methods 

### 2.1. Synthesis of 2S-Series Molecules

2 mmol 3-oxo-4-(triphenyl-λ^5^-phosphaneylidene)butanoate and 2 mmol aryl hydrazone compounds (1:1 molar ratio) were dissolved in approximately 30 mL of tetrahydrofuran. A catalytic amount of piperidine (2–3 drops) was added to the reaction mixture, which was stirred at room temperature for 24 h. Upon completion, the solvent was removed under reduced pressure using a rotary evaporator. The resulting residue was precipitated by the addition of methanol The crude product was filtered and purified by recrystallization from butanol.

In elucidating the structures of the synthesized compounds, techniques such as melting point, FTIR, ^1^H NMR, ^13^C NMR, and HR-MS were utilized. The FTIR spectra of the compounds were recorded using a Perkin Elmer Spectrum Two Model FT-IR spectrometer (Shelton, CT, USA), while the ^1^H- and ^13^C-NMR spectra were recorded using Bruker Advance 500 MHz and 400 MHz NMR instrument (Billerica, MA, USA) with DMSO-*d6* solvent. For HR-MS analyses, the Waters LCT Premier XE UPLC/MS TOF and Agilent Technologies 6224 TOF LC/MS instruments (Lexington, MA, USA) were used, and the Electrothermal 9200 instrument (Burladingen, Germany) was used for melting points. 

Ethyl (*E*)-2-(6-benzoyl-2-phenylpyridazine-3(2*H*)-ylidene)acetate (2S-1)

Mp 182–183 °C, Yields: %74, FT-IR (ATR, cm^−1^): 3098–2872 (aromatic and aliphatic C-H), 1672 (C=O, ester), 1646 (C=O, benzoyl), 1617–1460 (C=C and C=N). ^1^H-NMR (400 MHz; DMSO-*d*_6_, ppm): δ 8.69 (d, ^3^*J*_H-H_ = 10.1 Hz, 1H, CH, pyridazine), 7.92–7.42 (m, 11H, Ar–H), 4.33 (s, 1H, CH, aliphatic), 3.95 (q, 2H, ^3^*J*_H-H_ = 7.1 Hz, OCH_2_CH_3_), 1.08 (t, 3H, ^3^*J*_H-H_ = 7.1 Hz, OCH_2_CH_3_). ^13^C-NMR (100 MHz; DMSO-*d*_6_, ppm) (Sigma-Aldrich, St. Louis, MO, USA): δ 189.1 (CO, benzoyl), 166.9 (CO, ester), 150.1, 143.0, 142.8, 135.9, 133.4, 130.8, 130.7, 130.0, 129.3, 128.6, 126.7, 123.3 (C=C and C=N), 83.2 (CH, aliphatic), 58.8 (OCH_2_CH_3_), 14.8 (OCH_2_CH_3_). HRMS: *m*/*z* (M+H) calcd. for C_21_H_18_N_2_O_3_: 347.1390; found: 347.1393.

Ethyl (*E*)-2-(6-benzoyl-2-(4-methylphenyl)pyridazine-3(2*H*)-ylidene)acetate (2S-2)

Mp 149–150 °C, Yields: %76, FT-IR (ATR, cm^−1^): 3106–2891 ( C-H), 1676 (C=O, ester), 1638 (C=O, benzoyl), 1615–1446 (C=C and C=N). ^1^H-NMR (400 MHz; DMSO-*d*_6_, ppm): δ 8.66 (d, ^3^*J*_H-H_ = 10.1 Hz, 1H, CH, pyridazine), 7.91–7.37 (m, 10H, Ar–H), 4.33 (s, 1H, CH, aliphatic), 3.94 (q, 2H, ^3^*J*_H-H_ = 6.9 Hz, OCH_2_CH_3_), 2.36 (s, 3H, ArCH_3_), 1.08 (t, 3H, ^3^*J*_H-H_ = 7.0 Hz, OCH_2_CH_3_). ^13^C-NMR (100 MHz; DMSO-*d*_6_, ppm): δ 189.2 (CO, benzoyl), 166.9 (CO, ester), 150.2, 142.9, 140.4, 139.8, 135.9, 133.4, 131.2, 130.7, 129.2, 128.6, 126.4, 123.2 (C=C and C=N), 83.1 (CH, aliphatic), 58.9 (OCH_2_CH_3_), 21.2 (Ar-CH_3_), 14.8 (OCH_2_CH_3_). HRMS: *m*/*z* [M+H] calculated C_22_H_20_N_2_O_3_: 361.1547; found: 361.1548.

Ethyl (*E*)-2-(6-benzoyl-2-(2-methoxyphenyl)pyridazine-3(2*H*)-ylidene)acetate (2S-3)

Mp 184–185 °C, Yields: %78, FT-IR (ATR, cm^−1^): 3098–2843 (aromatic and aliphatic C-H), 1671 (C=O, ester), 1658 (C=O, benzoyl), 1608–1448 (C=C and C=N), 1260 (C-O). ^1^H-NMR (400 MHz; DMSO-*d*_6_, ppm): δ 8.66 (d, ^3^*J*_H-H_ = 10.1 Hz, 1H, CH, pyridazine), 7.89–7.12 (m, 10H, Ar–H), 4.18 (s, 1H, CH, aliphatic), 3.95 (bq, 2H, OCH_2_CH_3_), 3.85 (s, 3H, OCH_3_), 1.09 (t, 3H, ^3^*J*_H-H_ = 7.0 Hz, OCH_2_CH_3_). ^13^C-NMR (100 MHz; DMSO-*d*_6_, ppm): δ 189.1 (CO, benzoyl), 167.0 (CO, ester), 154.1, 149.9, 143.0, 135.9, 133.4, 131.9, 130.7, 130.6, 129.3, 128.6, 128.5, 123.2, 122.0, 114.0 (C=C and C=N), 82.6 (CH, aliphatic), 58.8 (OCH_2_CH_3_), 56.5 (OCH_3_), 14.8 (OCH_2_CH_3_). HRMS: *m*/*z* [M+H] calculated C_22_H_20_N_2_O_4_: 377.1496; found: 377.1507.

Ethyl (*E*)-2-(6-benzoyl-2-(4-fluorophenyl)pyridazine-3(2*H*)-ylidene)acetate (2S-4)

Mp 193–194 °C, Yields: %68, FT-IR (ATR, cm^−1^): 3102–2864 (aromatic and aliphatic C-H), 1669 (C=O, ester), 1657 (C=O, benzoyl), 1617–1444 (C=C and C=N). ^1^H-NMR (400 MHz; DMSO-*d*_6_, ppm): δ 8.68 (d, ^3^*J*_H-H_ = 10.1 Hz, 1H, CH, pyridazine), 7.92–7.42 (m, 10H, Ar–H), 4.29 (s, 1H, CH, aliphatic), 3.95 (q, 2H, ^3^*J*_H-H_ = 7.0 Hz, OCH_2_CH_3_), 1.09 (t, 3H, ^3^*J*_H-H_ = 7.1 Hz, OCH_2_CH_3_). ^13^C-NMR (100 MHz; DMSO-*d*_6_, ppm): δ 189.1 (CO, benzoyl), 166.8 (CO, ester), 162.4 (^1^*J*_C-F_= 298 Hz), 150.2, 143.0, 139.2, 135.9, 133.4, 130.7, 129.4, 129.3, 128.6, 123.3, 117.8 (^2^*J*_C-F_= 298 Hz) (C=C and C=N), 83.2 (CH, aliphatic), 58.9 (OCH_2_CH_3_), 14.8 (OCH_2_CH_3_). HRMS: *m*/*z* [M+H] calculated C_21_H_17_FN_2_O_3_: 365.1296; found: 365.1301.

Ethyl (*E*)-2-(6-benzoyl-2-(4-carbamoylphenyl)pyridazine-3(2*H*)-ylidene)acetate (2S-5)

Mp 222–223 °C, Yields: %72, FT-IR (ATR, cm^−1^): 3384, 3199 (NH_2_), 3088–2901 (aromatic and aliphatic C-H), 1665 (C=O, ester), 1652 (C=O, benzoyl), 1613–1445 (C=C and C=N). ^1^H-NMR (400 MHz; DMSO-*d*_6_, ppm): δ 8.68 (d, ^3^*J*_H-H_ = 10.1 Hz, 1H, CH, pyridazine), 8.11–7.43 (m, 12H, Ar–H ve NH_2_), 4.35 (s, 1H, CH, aliphatic), 3.96 (q, 2H, ^3^*J*_H-H_ = 7.0 Hz, OCH_2_CH_3_), 1.08 (t, 3H, ^3^*J*_H-H_ = 7.1 Hz, OCH_2_CH_3_). ^13^C-NMR (100 MHz; DMSO-*d*_6_, ppm): δ 189.2 (CO, benzoyl), 167.6 (CO, amide), 166.8 (CO, ester), 149.8, 144.9, 143.2, 135.8, 135.6, 133.5, 130.7, 130.0, 129.4, 128.6, 126.8, 123.2 (C=C and C=N), 83.5 (CH, aliphatic), 59.0 (OCH_2_CH_3_), 14.8 (OCH_2_CH_3_). HRMS: *m*/*z* [M+H] calculated C_22_H_19_N_3_O_4_: 390.1448; found: 390.1452.

Ethyl (*E*)-2-(6-benzoyl-2-(2-methylphenyl)pyridazine-3(2*H*)-ylidene)acetate (2S-6)

Mp 190–191 °C, Yields: %63, FT-IR (ATR, cm^−1^): 3086–2865 (aromatic and aliphatic C-H), 1678 (C=O, ester), 1660 (C=O, benzoyl), 1611–1448 (C=C and C=N). ^1^H-NMR (400 MHz; DMSO-*d*_6_, ppm): δ 8.69 (d, ^3^*J*_H-H_ = 10.1 Hz, 1H, CH, pyridazine), 7.88–7.43 (m, 10H, Ar–H), 4.06 (s, 1H, CH, aliphatic), 3.96 (bq, 2H, OCH_2_CH_3_), 2.15 (s, 3H, Ar-CH_3_), 1.09 (t, 3H, ^3^*J*_H-H_ = 6.6 Hz, OCH_2_CH_3_). ^13^C-NMR (100 MHz; DMSO-*d*_6_, ppm): δ 189.3 (CO, benzoyl), 167.0 (CO, ester), 149.6, 143.4, 141.3, 136.0, 134.4, 133.4, 132.3, 130.6, 130.5, 129.2, 128.7, 128.6, 127.4, 123.5 (C=C and C=N), 82.4 (CH, aliphatic), 58.9 (OCH_2_CH_3_), 17.0 (Ar-CH_3_), 14.8 (OCH_2_CH_3_). HRMS: *m*/*z* [M+H] calculated C_22_H_20_N_2_O_3_: 361.1547; found: 361.1544.

Etil (*E*)-2-(6-benzoyl-2-(4-klorofenil)pyridazine-3(2*H*)-iliden)asetat (2S-7)

Mp 155–156 °C, Yields: %69, FT-IR (ATR, cm^−1^): 3090–2873 (aromatic and aliphatic C-H), 1682 (C=O, ester), 1647 (C=O, benzoyl), 1620–1446 (C=C and C=N). ^1^H-NMR (400 MHz; DMSO-*d*_6_, ppm): δ 8.67 (d, ^3^*J*_H-H_ = 10.1 Hz, 1H, CH, pyridazine), 7.93–7.42 (m, 10H, Ar–H), 4.33 (s, 1H, CH, aliphatic), 3.96 (q, 2H, ^3^*J*_H-H_ = 7.0 Hz, OCH_2_CH_3_), 1.10 (t, 3H, ^3^*J*_H-H_ = 7.0 Hz, OCH_2_CH_3_). ^13^C-NMR (100 MHz; DMSO-*d*_6_, ppm): δ 189.1 (CO, benzoyl), 166.8 (CO, ester), 149.9, 143.1, 141.6, 135.8, 134.5, 133.4, 130.9, 130.7, 129.4, 128.8, 128.6, 123.2 (C=C and C=N), 83.4 (CH, aliphatic), 58.9 (OCH_2_CH_3_), 14.8 (OCH_2_CH_3_). HRMS: *m*/*z* [M+H] calculated C_21_H_17_ClN_2_O_3_: 381.1006; found: 381.1001.

Ethyl (*E*)-2-(6-benzoyl-2-(4-bromophenyl)pyridazine-3(2*H*)-ylidene)acetate (2S-8)

Mp 191–192 °C, Yields: %71, FT-IR (ATR, cm^−1^): 3095–2868 (aromatic and aliphatic C-H), 1687 (C=O, ester), 1636 (C=O, benzoyl), 1621–1450 (C=C and C=N). ^1^H-NMR (400 MHz; DMSO-*d*_6_, ppm): δ 8.67 (d, ^3^*J*_H-H_ = 10.1 Hz, 1H, CH, pyridazine), 7.92–7.43 (m, 10H, Ar–H), 4.31 (s, 1H, CH, aliphatic), 3.96 (q, 2H, ^3^*J*_H-H_ = 6.9 Hz, OCH_2_CH_3_), 1.10 (t, 3H, *J* = 7.0 Hz, OCH_2_CH_3_). ^13^C-NMR (100 MHz; DMSO-*d*_6_, ppm): δ 189.2 (CO, benzoyl), 166.8 (CO, ester), 149.9, 143.1, 142.0, 135.8, 133.9, 133.5, 130.7, 129.4, 129.1, 128.6, 123.2, 123.1 (C=C and C=N), 83.4 (CH, aliphatic), 59.0 (OCH_2_CH_3_), 14.8 (OCH_2_CH_3_). HRMS: *m*/*z* [M]-calculated C_21_H_17_BrN_2_O_3_: 424.0428; found: 424.0391.

Ethyl (*E*)-2-(6-(4-chlorobenzoyl)-2-phenylpyridazine-3(2*H*)-ylidene)acetate (2S-9)

Mp 219–220 °C, Yields: %79, FT-IR (ATR, cm^−1^): 3095–2933 (aromatic and aliphatic C-H), 1672 (C=O, ester), 1648 (C=O, benzoyl), 1619–1452 (C=C and C=N). ^1^H-NMR (400 MHz; DMSO-*d*_6_, ppm): δ 8.67 (d, ^3^*J*_H-H_ = 10.1 Hz, 1H, CH, pyridazine), 7.93–7.46 (m, 10H, Ar–H), 4.34 (s, 1H, CH, aliphatic), 3.94 (q, 2H, ^3^*J*_H-H_ = 7.0 Hz, OCH_2_CH_3_), 1.08 (t, 3H, ^3^*J*_H-H_ = 7.1 Hz, OCH_2_CH_3_). ^13^C-NMR (100 MHz; DMSO-*d*_6_, ppm): δ 188.0 (CO, benzoyl), 166.8 (CO, ester), 150.0, 142.9, 143.8, 138.4, 134.6, 132.6, 130.8, 130.0, 129.3, 128.7, 126.7, 123.1 (C=C and C=N), 83.5 (CH, aliphatic), 58.9 (OCH_2_CH_3_), 14.8 (OCH_2_CH_3_). HRMS: *m*/*z* [M+H] calculated C_21_H_17_ClN_2_O_3_: 381.1000; found: 381.1006.

Ethyl (*E*)-2-(6-(4-chlorobenzoyl)-2-(p-tolyl)pyridazine-3(2*H*)-ylidene)acetate (2S-10)

Mp 305–306 °C, Yields: %71, FT-IR (ATR, cm^−1^): 3117–2873 (aromatic and aliphatic C-H), 1668 (C=O, ester), 1645 (C=O, benzoyl), 1620–1452 (C=C and C=N). ^1^H-NMR (400 MHz; DMSO-*d*_6_, ppm): δ 8.66 (d, ^3^*J*_H-H_ = 10.0 Hz, 1H, CH, pyridazine), 7.93–7.41 (m, 9H, Ar–H), 4.34 (s, 1H, CH, aliphatic), 3.94 (q, 2H, ^3^*J*_H-H_ = 6.5 Hz, OCH_2_CH_3_), 2.50 (s, 3H, ArCH_3_), 1.09 (t, 3H, ^3^*J*_H-H_ = 6.8 Hz, OCH_2_CH_3_). ^13^C-NMR (100 MHz; DMSO-*d*_6_, ppm): δ 188.1 (CO, benzoyl), 166.9 (CO, ester), 150.1, 142.7, 140.3, 139.8, 138.3, 134.7, 132.5, 131.2, 129.2, 128.7, 126.4, 123.1 (C=C and C=N), 83.4 (CH, aliphatic), 58.9 (OCH_2_CH_3_), 21.2 (ArCH_3_), 14.8 (OCH_2_CH_3_). HRMS: *m*/*z* [M+H] calculated C_22_H_19_ClN_2_O_3_: 395.1157; found: 395.1154.

Ethyl (*E*)-2-(6-(4-chlorobenzoyl)-2-(2-methoxyphenyl)pyridazine-3(2*H*)-ylidene)acetate (2S-11)

Mp 205–206 °C, Yields: %71, FT-IR (ATR, cm^−1^): 3092–2845 (aromatic and aliphatic C-H), 1667 (C=O, ester), 1651 (C=O, benzoyl), 1616–1464 (C=C and C=N). ^1^H-NMR (400 MHz; DMSO-*d*_6_, ppm): δ 8.65 (d, ^3^*J*_H-H_ = 10.1 Hz, 1H, CH, pyridazine), 7.90–7.12 (m, 9H, Ar–H), 4.18 (s, 1H, CH, aliphatic), 3.95 (q, 2H, ^3^*J*_H-H_ = 6.5 Hz, OCH_2_CH_3_), 3.85 (s, 3H, OCH_3_), 1.09 (t, 3H, ^3^*J*_H-H_ = 7.0 Hz, OCH_2_CH_3_). ^13^C-NMR (100 MHz; DMSO-*d*_6_, ppm): δ 187.9 (CO, benzoyl), 167.0 (CO, ester), 154.0, 149.8, 142.8, 138.4, 134.5, 132.5, 132.0, 130.5, 129.3, 128.7, 128.4, 123.1, 122.1, 114.0 (C=C and C=N), 82.9 (CH, aliphatic), 58.9 (OCH_2_CH_3_), 56.5 (OCH_3_), 14.8 (OCH_2_CH_3_). HRMS: *m*/*z* [M+H] calculated C_22_H_19_ClN_2_O_4_: 411.1106; found: 411.1113.

Ethyl (*E*)-2-(6-(4-chlorobenzoyl)-2-(4-fluorophenyl)pyridazine-3(2*H*)-ylidene)acetate (2S12)

Mp 193–194 °C, Yields: %68, FT-IR (ATR, cm^−1^): 3105–2871 (aromatic and aliphatic C-H), 1669 (C=O, ester), 1651 (C=O, benzoyl), 1619–1445 (C=C and C=N). ^1^H-NMR (400 MHz; DMSO-*d*_6_, ppm): δ 8.67 (d, ^3^*J*_H-H_ = 10.1 Hz, 1H, CH, pyridazine), 7.92–7.42 (m, 9H, Ar–H), 4.34 (s, 1H, CH, aliphatic), 3.96 (q, 2H, ^3^*J*_H-H_ = 7.0 Hz, OCH_2_CH_3_), 1.10 (t, 3H, ^3^*J*_H-H_ = 7.0 Hz, OCH_2_CH_3_). ^13^C-NMR (100 MHz; DMSO-*d*_6_, ppm): δ 188.01 (CO, benzoyl), 166.8 (CO, ester), 162.4 (^1^*J*_C-F_= 247.5 Hz), 150.1, 142.8, 139.1, 138.4, 134.6, 132.6, 129.3, 129.2, 128.7, 123.1, 117.8 (^2^*J*_C-F_= 23.1 Hz) (C=C and C=N), 83.5 (CH, aliphatic), 58.9 (OCH_2_CH_3_), 14.8 (OCH_2_CH_3_). HRMS: *m*/*z* [M+H] calculated C_21_H_16_ClFN_2_O_3_: 399.0906; found: 399.0906.

Ethyl (*E*)-2-(2-(4-carbamoylphenyl)-6-(4-chlorobenzoyl)pyridazine-3(2*H*)-ylidene)acetate (2S-13)

Mp 310–311 °C, Yields: %70, FT-IR (ATR, cm^−1^): 3382, 3302 (NH_2_), 3168–2867 (aromatic and aliphatic C-H), 1688 (C=O, ester), 1650 (C=O, benzoyl), 1641 (C=O, amide), 1615–1485 (C=C and C=N). ^1^H-NMR (400 MHz; DMSO-*d*_6_, ppm): δ 8.67 (d, 1H, *J* = 10.1 Hz, CH), 8.13–7.47 (m, 11H, Ar–H, NH_2_), 4.36 (s, 1H, CH), 3.95 (q, 2H, *J* = 7.0 Hz, OCH_2_CH_3_), 1.09 (t, 3H, *J* = 7.1 Hz, OCH_2_CH_3_). ^13^C-NMR (100 MHz; DMSO-*d*_6_, ppm): δ 187.9 (CO, benzoyl), 167.4 (CO, amide), 166.8 (CO, ester), 149.7, 144.8, 143.0, 138.4, 135.7, 134.6, 132.6, 130.0, 129.4, 128.7, 126.7, 123.0 (C=C and C=N), 83.8 (CH, aliphatic), 59.0 (OCH_2_CH_3_), 14.8 (OCH_2_CH_3_). HRMS: *m*/*z* [M+H] calculated C_22_H_18_ClN_3_O_4_: 424.1059; found: 424.1056.

Ethyl (*E*)-2-(6-(4-chlorobenzoyl)-2-(o-tolyl)pyridazine-3(2*H*)-ylidene)acetate (2S-14)

Mp 199–200 °C, Yields: %68, FT-IR (ATR, cm^−1^): 3096–2868 (aromatic and aliphatic C-H), 1669 (C=O, ester), 1661 (C=O, benzoyl), 1618–1459 (C=C and C=N). ^1^H-NMR (400 MHz; DMSO-*d*_6_, ppm): δ 8.68 (d, ^3^*J*_H-H_ = 10.1 Hz, 1H, CH, pyridazine), 7.88–7.44 (m, 9H, Ar–H), 4.04 (s, 1H, CH, aliphatic), 3.94 (q, 2H, ^3^*J*_H-H_ = 6.0 Hz, OCH_2_CH_3_), 2.15 (s, 3H, Ar-CH_3_), 1.09 (t, 3H, ^3^*J*_H-H_ = 7.1 Hz, OCH_2_CH_3_). ^13^C-NMR (100 MHz; DMSO-*d*_6_, ppm): δ 188.2 (CO, benzoyl), 167.0 (CO, ester), 149.6, 143.2, 141.4, 141.2, 138.3, 134.7, 134.4, 132.5, 132.4, 132.3, 130.5, 129.2, 128.8, 127.4, 123.4 (C=C and C=N), 82.6 (CH, aliphatic), 17.0 (ArCH_3_), 14.8 (OCH_2_CH_3_). HRMS: *m*/*z* [M+H] calculated C_22_H_19_ClN_2_O_3_: 395.1157; found: 395.1159.

Ethyl (*E*)-2-(6-(4-chlorobenzoyl)-2-(4-chlorophenyl)pyridazine-3(2*H*)-ylidene)acetate (2S-15)

Mp 206–207 °C, Yields: %77, FT-IR (ATR, cm^−1^): 3069–2830 (aromatic and aliphatic C-H), 1678 (C=O, ester), 1651 (C=O, benzoyl), 1605–1457 (C=C and C=N). ^1^H-NMR (400 MHz; DMSO-*d*_6_, ppm): δ 8.67 (d, ^3^*J*_H-H_ = 10.1 Hz, 1H, CH, pyridazine), 7.94–7.44 (m, 9H, Ar–H), 4.37 (s, 1H, CH, aliphatic), 3.96 (bq, 2H, OCH_2_CH_3_), 1.10 (t, 3H, *J* = 6.4 Hz, OCH_2_CH_3_). ^13^C-NMR (100 MHz; DMSO-*d*_6_, ppm): δ 187.9 (CO, benzoyl), 166.8 (CO, ester), 149.8, 142.9, 141.5, 138.4, 134.5, 134.5, 132.5, 130.9, 129.4, 128.7, 128.7, 123.0 (C=C and C=N), 83.7 (CH, aliphatic), 59.0 (OCH_2_CH_3_), 14.7 (OCH_2_CH_3_). HRMS: m/z [M+H] calculated C_21_H_16_Cl_2_N_2_O_3_: 415.0611; found: 415.0611.

Ethyl (*E*)-2-(2-(4-bromophenyl)-6-(4-chlorobenzoyl)pyridazine-3(2*H*)-ylidene)acetate (2S-16)

Mp 172–173 °C, Yields: %69, FT-IR (ATR, cm^−1^): 3092–2872 (aromatic and aliphatic C-H), 1678 (C=O, ester), 1651 (C=O, benzoyl), 1607–1448 (C=C and C=N). ^1^H-NMR (400 MHz; DMSO-*d*_6_, ppm): δ 8.66 (d, ^3^*J*_H-H_ = 10.1 Hz, 1H, CH, pyridazine), 7.93–7.45 (m, 9H, Ar–H), 4.34 (s, 1H, CH, aliphatic), 3.96 (q, 2H, ^3^*J*_H-H_ = 6.9 Hz, OCH_2_CH_3_), 1.09 (t, 3H, ^3^*J*_H-H_ = 7.0 Hz, OCH_2_CH_3_). ^13^C-NMR (100 MHz; DMSO-*d*_6_, ppm): δ 188.0 (CO, benzoyl), 166.8 (CO, ester), 149.8, 143.0, 141.9, 138.4, 134.5, 133.9, 132.6, 129.4, 129.0, 128.7, 123.1, 123.0 (C=C and C=N), 83.7 (CH, aliphatic), 59.0 (OCH_2_CH_3_), 14.8 (OCH_2_CH_3_).

Ethyl (*E*)-2-(6-(4-methylbenzoyl)-2-phenylpyridazine-3(2*H*)-ylidene)acetate (2S-17)

Mp 193–194 °C, Yields: %69, FT-IR (ATR, cm^−1^): 3104–2869 (aromatic and aliphatic C-H), 1679 (C=O, ester), 1637 (C=O, benzoyl), 1619–1453 (C=C and C=N). ^1^H-NMR (400 MHz; DMSO-*d*_6_, ppm): δ 8.67 (d, 1H, ^3^*J*_H-H_ = 10.1 Hz, 1H, CH, pyridazine), 7.83 (d, Part A of AA’BB’ system, ^3^*J*_H-H_ = 8.1 Hz, 2H, ArH,), 7.62–7.45 (m, 6H, Ar–H), 7.24 (d, Part B of AA’BB’ system, ^3^*J*_H-H_ = 8.1 Hz, 2H, ArH), 4.31 (s, 1H, CH, aliphatic), 3.94 (q, 2H, ^3^*J*_H-H_ = 7.1 Hz, OCH_2_CH_3_), 2.31 (s, 3H, Ar-CH_3_), 1.08 (t, 3H, ^3^*J*_H-H_ = 7.1 Hz, OCH_2_CH_3_). ^13^C-NMR (100 MHz; DMSO-*d*_6_, ppm): δ 188.7 (CO, benzoyl), 166.9 (CO, ester), 150.1, 144.0, 143.8, 142.9, 133.2, 130.9, 130.8, 130.0, 129.3, 129.2, 126.8, 123.4 (C=C and C=N), 83.0 (CH, aliphatic), 58.8 (OCH_2_CH_3_), 21.6 (Ar-CH_3_), 14.8 (OCH_2_CH_3_). HRMS: *m*/*z* [M+H] calculated C_22_H_20_N_2_O_3_: 361.1547; found: 361.1548.

Ethyl (*E*)-2-(6-(4-methylbenzoyl)-2-(p-tolyl)-pyridazine-3(2*H*)-ylidene)acetate (2S-18)

Mp 178–179 °C, Yields: %73, FT-IR (ATR, cm^−1^): 3098–2894 (aromatic and aliphatic C-H), 1681 (C=O, ester), 1639 (C=O, benzoyl), 1615–1454 (C=C and C=N). ^1^H-NMR (400 MHz; DMSO-*d*_6_, ppm): δ 8.66 (d, 1H, ^3^*J*_H-H_ = 10.1 Hz, CH, pyridazine), 7.83–7.24 (m, 9H, Ar–H), 4.31 (s, 1H, CH, aliphatic), 3.94 (q, 2H, ^3^*J*_H-H_ = 6.9 Hz, OCH_2_CH_3_), 2.37 (s, 3H, Ar-CH_3_), 2.32 (s, 3H, Ar-CH_3_), 1.09 (t, 3H, ^3^*J*_H-H_ = 7.0 Hz, OCH_2_CH_3_). ^13^C-NMR (100 MHz; DMSO-*d*_6_, ppm): δ 188.7 (CO, benzoyl), 166.9 (CO, ester), 150.2, 144.0, 143.0, 140.4, 139.8, 133.2, 131.2, 131.0, 130.9, 129.2, 126.5, 123.4 (C=C and C=N), 82.9 (CH, aliphatic), 58.8 (OCH_2_CH_3_), 21.6 (ArCH_3_), 21.2 (ArCH_3_), 14.8 (OCH_2_CH_3_). HRMS: *m*/*z* [M+H] calculated C_23_H_22_N_2_O_3_: 375.1703; found: 375.1709.

Ethyl (*E*)-2-(2-(2-methoxyphenyl)-6-(4-methylbenzoyl)pyridazine-3(2*H*)-ylidene)acetate (2S-19)

Mp 171–172 °C, Yields: %72, FT-IR (ATR, cm^−1^): 3094–2838 (aromatic and aliphatic C-H), 1672 (C=O, ester), 1656 (C=O, benzoyl), 1646–1456 (C=C and C=N), 1260 (C-O). ^1^H-NMR (400 MHz; DMSO-*d*_6_, ppm): δ 8.69 (d, 1H, ^3^*J*_H-H_ = 10.1 Hz, CH, pyridazine), 7.81–7.14 (m, 9H, Ar–H), 4.16 (s, 1H, CH, aliphatic), 3.94 (bq, 2H, OCH_2_CH_3_), 3.84 (s, 3H, OCH_3_), 1.08 (t, 3H, ^3^*J*_H-H_ = 7.1 Hz, OCH_2_CH_3_). ^13^C-NMR (100 MHz; DMSO-*d*_6_, ppm): δ 188.6 (CO, benzoyl), 167.0 (CO, ester), 154.1, 149.9, 144.1, 143.2, 133.2, 131.9, 130.9, 130.8, 130.7, 129.2, 128.5, 123.3, 122.0, 114.0 (C=C and C=N), 82.4 (CH, aliphatic), 58.8 (OCH_2_CH_3_), 56.5 (OCH_3_), 21.5 (Ar-CH_3_), 14.8 (OCH_2_CH_3_). HRMS: *m*/*z* [M+H] calculated C_23_H_22_N_2_O_4_: 391.1613; found: 391.1648.

Ethyl (*E*)-2-(2-(4-fluorophenyl)-6-(4-methylbenzoyl)pyridazine-3(2*H*)-ylidene)acetate (2S-20)

Mp 191–192 °C, Yields: %75, FT-IR (ATR, cm^−1^): 3099–2901 (aromatic and aliphatic C-H), 1681 (C=O, ester), 1639 (C=O, benzoyl), 1618–1447 (C=C and C=N). ^1^H-NMR (400 MHz; DMSO-*d*_6_, ppm): δ 8.67 (d, 1H, ^3^*J*_H-H_ = 10.1 Hz, CH, pyridazine), 7.84–7.24 (m, 9H, Ar–H), 4.29 (s, 1H, CH, aliphatic), 3.96 (q, 2H, ^3^*J*_H-H_ = 6.9 Hz, OCH_2_CH_3_), 2.32 (s, 3H, ArCH_3_), 1.10 (t, 3H, ^3^*J*_H-H_ = 7.0 Hz, OCH_2_CH_3_). ^13^C-NMR (100 MHz; DMSO-*d*_6_, ppm): δ 188.7 (CO, benzoyl), 166.9 (CO, ester), 162.4 (d, ^1^*J*_C-F_= 246 Hz), 150.2, 144.1, 143.2, 139.2, 133.2, 130.9, 129.4, 129.3, 129.2, 123.4, 117.8 (d, ^2^*J*_C-F_= 23 Hz) (C=C and C=N), 83.1 (CH, aliphatic), 58.9 (OCH_2_CH_3_), 21.6 (Ar-CH_3_), 14.8 (OCH_2_CH_3_). HRMS: *m*/*z* [M+H] calculated C_22_H_19_FN_2_O_3_: 379.1452; found: 379.1418.

Ethyl (*E*)-2-(2-(4-fluorophenyl)-6-(4-methylbenzoyl)pyridazine-3(2*H*)-ylidene)acetate (2S-21)

Mp 237–238 °C, Yields: %73, FT-IR (ATR, cm^−1^): 3413, 3205 (NH_2_), 3105–2904 (aromatic and aliphatic C-H), 1684 (C=O, amide), 1665 (C=O, ester), 1646 (C=O, benzoyl), 1619–1446 (C=C and C=N). ^1^H-NMR (400 MHz; DMSO-*d*_6_, ppm): δ 8.68 (d, 1H, ^3^*J*_H-H_ = 10.1 Hz, CH, pyridazine), 8.12 ve 7.54 (s, 2H, NH_2_), 8.05 (d, Part B of AA’BB’ system, ^3^*J*_H-H_ = 8.3 Hz, 2H, ArH), 7.84 (d, Part A of AA’BB’ system, ^3^*J*_H-H_ = 8.0 Hz, 2H, ArH), 7.67 (d, Part A of AA’BB’ system, ^3^*J*_H-H_ = 8.3 Hz, 2H, ArH), 7.47 (d, ^3^*J*_H-H_ = 10.1 Hz, 1H, CH, pyridazine), 7.26 (d, Part B of AA’BB’ system, ^3^*J*_H-H_ = 7.9 Hz, 2H, ArH), 4.34 (s, 1H, CH, aliphatic), 3.95 (q, 2H, ^3^*J*_H-H_ = 7.0 Hz, OCH_2_CH_3_), 2.32 (s, 3H, ArCH_3_), 1.09 (t, 3H, ^3^*J*_H-H_ = 7.0 Hz, OCH_2_CH_3_). ^13^C-NMR (100 MHz; DMSO-*d*_6_, ppm): δ 188.7 (CO, benzoyl), 167.4 (CO, amide), 166.8 (CO, ester), 149.8, 144.9, 144.0, 143.3, 135.7, 133.2, 130.9, 130.0, 129.4, 129.2, 126.8, 123.4 (C=C and C=N), 83.3 (CH, aliphatic), 58.9 (OCH_2_CH_3_), 21.6 (Ar-CH_3_), 14.8 (OCH_2_CH_3_). HRMS: *m*/*z* [M+H] calculated C_23_H_21_N_3_O_4_: 404.1605; found: 404.1606.

Ethyl (*E*)-2-(6-(4-methylbenzoyl)-2-(2-methylphenyl)pyridazine-3(2*H*)-ylidene)acetate (2S-22)

Mp 166–167 °C, Yields: %64, FT-IR (ATR, cm^−1^): 3094–2837 (aromatic and aliphatic C-H), 1672 (C=O, ester), 1654 (C=O, benzoyl), 1644–1454 (C=C and C=N). ^1^H-NMR (400 MHz; DMSO-*d*_6_, ppm): δ 8.66 (d, ^3^*J*_H-H_ = 10.1 Hz, 1H, CH, pyridazine), 7.82–7.12 (m, 9H, Ar-H), 4.17 (s, 1H, CH, aliphatic), 3.95 (bq, 2H, OCH_2_CH_3_), 3.85 (s, 3H, Ar-CH_3_), 2.32 (s, 3H, Ar-CH_3_), 1.09 (t, 3H, ^3^*J*_H-H_ = 7.0 Hz, OCH_2_CH_3_). ^13^C-NMR (100 MHz; DMSO-*d*_6_, ppm): δ 188.6 (CO, benzoyl), 167.0 (CO, ester), 154.1, 149.9, 144.1, 143.2, 133.2, 131.9, 130.9, 130.8, 130.7, 129.2, 128.5, 123.3, 122.0, 114.0 (C=C and C=N), 82.4 (CH, aliphatic), 58.8 (OCH_2_CH_3_), 56.5 (Ar-CH_3_), 21.5 (Ar-CH_3_), 14.8 (OCH_2_CH_3_). HRMS: *m*/*z* [M+H] calculated C_23_H_22_N_2_O_3_: 375.1703; found: 375.1709.

Ethyl (*E*)-2-(2-(4-chlorophenyl)-6-(4-methylbenzoyl)pyridazine-3(2*H*)-ylidene)acetate (2S-23)

Mp 174–175 °C, Yields: %76, FT-IR (ATR, cm^−1^): 3094–2898 (aromatic and aliphatic C-H), 1682 (C=O, ester), 1645 (C=O, benzoyl), 1607–1446 (C=C and C=N). ^1^H-NMR (400 MHz; DMSO-*d*_6_, ppm): δ 8.67 (d, 1H, ^3^*J*_H-H_ = 10.1 Hz, CH), 7.44 (d, 1H, ^3^*J*_H-H_ = 10.1 Hz, CH), 7.85–7.24 (m, 8H, Ar–H), 4.33 (s, 1H, CH, aliphatic), 3.96 (q, 2H, ^3^*J*_H-H_ = 6.9 Hz, OCH_2_CH_3_), 2.32 (s, 3H, Ar-CH_3_), 1.10 (t, 3H, ^3^*J*_H-H_ = 7.0 Hz, OCH_2_CH_3_). ^13^C-NMR (100 MHz; DMSO-*d*_6_, ppm): δ 188.6 (CO, benzoyl), 166.8 (CO, ester), 149.9, 144.0, 143.3, 141.6, 134.5, 133.2, 130.9, 130.9, 129.4, 129.2, 128.9, 123.3 (C=C and C=N), 83.2 (CH, aliphatic), 58.9 (OCH_2_CH_3_), 21.6 (Ar-CH_3_), 14.8 (OCH_2_CH_3_). HRMS: *m*/*z* [M+H] calculated C_22_H_19_ClN_2_O_3_: 395.1157; found: 395.1163.

Ethyl (*E*)-2-(2-(4-bromophenyl)-6-(4-methylbenzoyl)pyridazine-3(2*H*)-ylidene)acetate (2S-24)

Mp 193–194 °C, Yields: %72, FT-IR (ATR, cm^−1^): 3093–2870 (aromatic and aliphatic C-H), 1682 (C=O, ester), 1647 (C=O, benzoyl), 1608–1448 (C=C and C=N). ^1^H-NMR (400 MHz; DMSO-*d*_6_, ppm): δ 8.66 (d, 1H, ^3^*J*_H-H_ = 10.1 Hz, CH), 7.84–7.24 (m, 9H, Ar–H), 4.32 (s, 1H, CH, aliphatic), 3.96 (q, 2H, ^3^*J*_H-H_ = 6.3 Hz, OCH_2_CH_3_), 2.33 (s, 3H, Ar-CH_3_), 1.10 (t, 3H, ^3^*J*_H-H_ = 6.8 Hz, OCH_2_CH_3_). ^13^C-NMR (100 MHz; DMSO-*d*_6_, ppm): δ 188.7 (CO, benzoyl), 166.8 (CO, ester), 149.9, 144.1, 143.3, 142.0, 133.9, 133.1, 130.9, 129.4, 129.2, 129.1, 123.4, 123.0 (C=C and C=N), 83.2 (CH, aliphatic), 58.9 (OCH_2_CH_3_), 21.6 (Ar-CH_3_), 14.8 (OCH_2_CH_3_). HRMS: *m*/*z* [M+H] calculated C_22_H_19_BrN_2_O_3_: 439.0652; found: 439.0652.

### 2.2. Cell Culture

MDA-MB-231 and 4T1 were selected as representative models of human and mouse TNBC, respectively, due to their well-characterized aggressive phenotypes and widespread use in preclinical studies. Mouse 4T1 is widely used for in vivo metastasis models, as it mimics human TNBC progression and the immune environment. It provides comparative insights between human and murine TNBC biology. The hTERT-immortalized breast epithelial cell line was used to evaluate selectivity and cytotoxicity against healthy cells.

Commercially available 4T1 (CRL-2539), MDA-MB-231 (HTB-26), and hTERT (CRL-4010) cell lines (The American Type Culture Collection: ATCC, Manassas, VA, USA) were incubated at 37 °C, with 5% CO_2_, and cultured in a medium containing 10% fetal bovine serum (FBS; Sigma-Aldrich, Burlington, MA, USA), high-glucose Dulbecco modified Eagle’s medium (DMEM; Euroclone S.p.A., Pero, Italy), and 1% penicillin–streptomycin (10,000 U/mL; Capricorn Scientific, Ebsdorfergrund, Germany) in an environment with 95% humidity. Cells that reached 80–90% density were washed with Dulbecco’s phosphate-buffered saline (DPBS; Euroclone S.p.A., Pero, Italy) and detached from the surface using 0.25% Trypsin-EDTA (Sigma-Aldrich, Burlington, MA, USA). The trypsin–EDTA–cell mixture was centrifuged at 1200 rpm for 3 min. After centrifugation, the supernatant was removed, and fresh medium was added. Cells were seeded onto 96-well plates (Corning Inc., Corning, NY, USA) (7 × 10^3^ cells/well) and incubated.

### 2.3. Dissolving 2S-Series Molecules

The synthesized 2S-series molecules were weighed according to their molecular weight. The substances were dissolved with DMSO so that the main stock was 100 mM. Intermediate stocks were prepared by dilution with ddH_2_O to 100 µM and stored at −20 °C to be used in experiments.

### 2.4. MTT Tests

#### 2.4.1. Determination of Anticancer Activities of 2S-Series Molecules

The purpose of these experiments was to determine the cytotoxic effects of 2S-series compounds and compare their potency with that of the chemotherapeutic agent doxorubicin (DOX) using a colorimetric MTT viability assay.

For viability experiments of molecules in cell lines, the 4T1, MDA-MB-231, and hTERT cell lines were removed with Trypsin-EDTA (Sigma-Aldrich) when they reached sufficient cell density in culture dishes. They were seeded in 96-well cell plates with 7 × 10^3^ cells per 100 µL in each well. After the cells adhered to the plates 24 h, the dissolved 2S-series molecules (1–24) were applied to the wells at 20 µM. After 48 h, the medium containing the inhibitors was removed and the cells were washed with PBS (Euroclone S.p.A.). After adding 100 µL DMEM (Euroclone S.p.A.) and 20 µL MTT solution (T0793(DB0362), Bio Basic Inc., Markham, ON, Canada) to each well, the plates were incubated for 3 h. At the end of the incubation, absorbances were measured in an ELISA reader (Multiskan™ GO; Thermo Fisher Scientific, Waltham, MA, USA) at a wavelength of 570 nm.

#### 2.4.2. Determination of Anticancer Activities of 2S-Series Molecules

The anticancer activity of 2S-5 and 2S-13 inhibitors in the 4T1, MDA-MB-231, and hTERT cell lines was assessed using MTT assays at 48 and 72 h (n = 5). Cells were seeded onto 96-well plates at a density of 7 × 10^3^ cells per well in a 100 µL medium. This seeding density was chosen based on preliminary optimization to ensure that cells remained in the exponential growth phase during compound exposure and to prevent confluency over the 72 h incubation period. After 24 h of attachment, the compounds were applied at concentrations of 3.125, 6.25, 12.5, 25, 50, and 100 µM. Following 48 or 72 h of incubation, 20 µL of MTT solution (5 mg/mL) was added and incubated for 3 h. Absorbance values were measured at 570 nm using an ELISA reader (Multiskan GO, Thermo). The IC50 values were calculated using GraphPad Prism 9 software (GraphPad Software, San Diego, CA, USA) Selectivity index (SI) values were determined as the ratio of the IC50 in hTERT cells to that in cancer cells (4T1 and MDA-MB-231).

#### 2.4.3. Doxorubicin (DOX) Application

To determine the IC_50_ value of the DOX, 4T1, MDA-MB-231, and hTERT lines were seeded onto 96-well plates at 7 × 10^3^ cells/well. After the cells adhered to the wells for 24 h, DOX was incubated for 48 h at concentrations of 50–25–12.5–6.25–3.125–1.56–0.781–0.390–0.195–0.097–0.048–0.024 µM. The cell group in which inhibitors were not applied was considered the control group. At the end of the experiment period, the medium was removed from all wells and 100 μL of medium and 20 μL (5 mg/mL) MTT solution were added to the wells. After 3 h of incubation, 100 μL of DMSO was added to each well. Absorbance values were measured in an ELISA reader at 570 nm. Results were determined as the 2S-series.

### 2.5. RT-QPCR Array

The aim of the RT-qPCR analysis was to determine the expression changes in genes associated with cancer-related signaling pathways in response to treatment with DOX and 2S-13.

The total RNA extractions were performed with Innuprep Rna Mini Kit 2.0 Analytik Jena and via the synthesis of cDNA with 1000 ng of total RNA using ABM Good cDNA Synthesis Kit. For real-time q-PCR, active genes in human and mouse cancer cell pathways were examined in the cells before and after drug application, and the drug effect was examined at the gene expression level. The amount of DNA was determined after each replication by binding the fluorescent dye in the PCR mixture to the DNA, thus giving a C_T_ value. An in-house PCR array was employed to determine the pathways in cancer signaling. Alterations were observed in the array genes employed to determine the molecular mechanism associated. RT-QPCR analyses were performed using the in-house array primers (Sigma-Aldrich) on each cDNA, using SYBR Green Master Mix (FluoCycle II SYBR Master Mix 2X) and GAPDH as the reference gene. In this study, GAPDH and B2M were used for normalization and the relative expression levels of genes were calculated using the 2^−ΔΔCt^ method. Analyses of the results were completed using the Enrich database.

### 2.6. Cell Cycle Analysis

The analysis was carried out in accordance with the instructions of the MAK344 Cell Cycle Analysis kit. MDA-MB-231 and 4T1 cells (6 × 10^5^ cells/well) were treated with 2S-13 and DOX and prepared with the kit protocol. Then, the cells were analyzed using Flow cytometry (CytoFLEX, Beckman Coulter, Brea, CA, USA).

### 2.7. Apoptosis Analysis

The apoptosis assay was used to quantify early and late apoptotic events triggered by 2S-13 and DOX, providing insights into the mechanism of cell death. The analysis was carried out in accordance with the instructions of the Annexin V FITC—Apoptosis Detection Kit. MDA-MB-231 and 4T1 cells (6 × 10^5^ cells/well) treated were with 2S-13 and DOX and prepared with the kit protocol. Then, the cells were analyzed using flow cytometry (Beckman coulter, Cytoflex).

### 2.8. Molecular Docking Analysis

The molecular structures of the 2S-5 and 2S-13 ligands were drawn using MarvinSketch (ChemAxon^®^) and saved in SDF format. The 3D conformer of doxorubicin (DOX) was obtained from the PubChem database. The crystal structure of the human Hsp90α N-terminal domain (NTD) was retrieved from the Protein Data Bank (PDB ID: 3T0Z).

Molecular docking simulations were conducted using YASARA Structure software (https://www.yasara.org/ accessed on 1 September 2025) with the AutoDock-based algorithm. Protein preparation involved the removal of crystallographic water molecules, the addition of polar hydrogen atoms, the adjustment of protonation states to physiological pH, and energy minimization. To validate the docking protocol, ATP was re-docked into its native binding pocket within the Hsp90α NTD, and the predicted pose was compared with the co-crystallized reference conformation.

For each ligand (DOX, 2S-5, and 2S-13), 25 docking poses were generated. The pose with the lowest (most negative) binding energy was selected for further analysis. Binding energies (in kcal/mol) and corresponding dissociation constants (KD values) were calculated to assess and compare the binding affinities of the compounds to the Hsp90α NTD.

## 3. Results

### 3.1. Synthesis and Characterization of 2S-Series

**Scheme 1:**
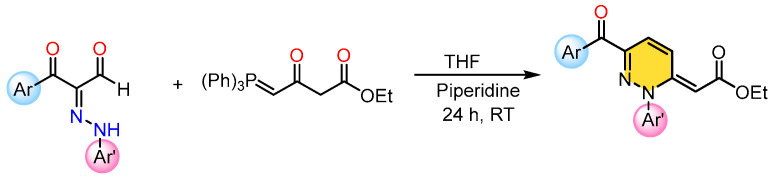
Synthesis reaction of target pyridazines.

The 2S-series compounds were obtained through the reaction of related aryl hydrazone derivatives with ethyl 3-oxo-4-(triphenyl-λ^5^-phosphaneylidene)butanoate (Scheme 1). The structure of the synthesized compounds was thoroughly elucidated using spectroscopic techniques including FT-IR, ^1^H NMR, ^13^C NMR, and HRMS analyses.

Molecular formulas, melting points, reaction yields and HRMS analysis data of the synthesized and characterized 2S compounds are given in Table 1. Derivatization of target compounds was achieved by attaching different substituents to the benzoyl and diazo groups. The benzoyl termini of the molecules were modified with acetophenone, 4-chloroacetophenone, and 4-methylacetophenone reagents, while the diazo termini were modified with seven different aromatic amine derivatives. Target compounds with different electronic environments were obtained by combining the halogen, methyl, methoxy, and benzamide groups found in the aromatic amine derivatives. Modifying molecules with electron-donating and electron-accepting groups has been an important factor in the preference of compounds based on their structure–activity relationships.

As a representative example, the characterization of compound 2S-1 is presented in this section. Following purification procedures after the reaction, compound 2S-1 was isolated with a yield of 74%. Furthermore, the calculated and observed HRMS values for [M+H]^+^ were in good agreement, confirming the proposed molecular formula (C_21_H_18_N_2_O_3_) of the compound. The FT-IR spectrum of compound 2S-1, recorded using the ATR technique, exhibits absorption bands in the range of 3098–2872 cm^−1^, which are attributed to aromatic and aliphatic C–H stretching vibrations. The bands observed at 1672 and 1646 cm^−1^ correspond to the stretching vibrations of carbonyl (C=O) groups. Characteristic stretching vibrations of aromatic C=C bonds are observed in the region of 1617–1460 cm^−1^. In the ^1^H NMR spectrum of compound 2S-1, recorded in DMSO-_d6_, a doublet signal at 8.69 ppm is assigned to the proton on the pyridazine ring. Multiplet signals between 7.92 and 7.42 ppm correspond to the remaining aromatic protons, including those on the pyridazine moiety, representing a total of 11 aromatic protons. A singlet at 4.33 ppm is attributed to the olefinic =CH proton, while a quartet at 3.95 ppm is attributed to the ester –OCH_2_ protons. A triplet at 1.08 ppm corresponds to the –CH_3_ protons. In the ^13^C NMR spectrum of compound 2S-1, recorded in DMSO-_d6_, a signal at 189.1 ppm is attributed to the benzoyl carbonyl carbon, and the signal at 166.9 ppm corresponds to the ester carbonyl carbon. Signals observed in the range of 150.1–123.3 ppm are assigned to twelve carbon atoms belonging to aromatic C=C and heteroaromatic C=N groups. In addition, the olefinic carbon appears at 83.2 ppm, the –OCH_2_ group at 58.8 ppm, and the –CH_3_ group at 14.8 ppm.

### 3.2. Effect of 2S-Series Molecules on 4T1 and MDA-MB-231 Cell Line

**Figure 1:**
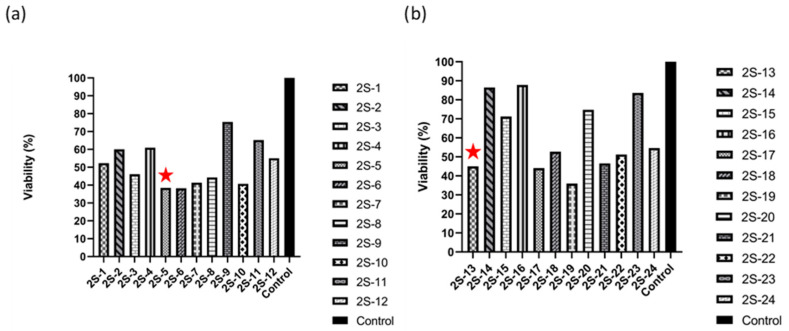
(**a**) Viability percentages of 2S-series molecules (1–12) in 4T1 cell line; (**b**) viability percentages of 2S-series molecules (13–24) in 4T1 cell line.

Figure 1 illustrates the cell viability results of the 2S-series compounds (2S-1 to 2S-24) in the 4T1 cell line.

**Figure 2:**
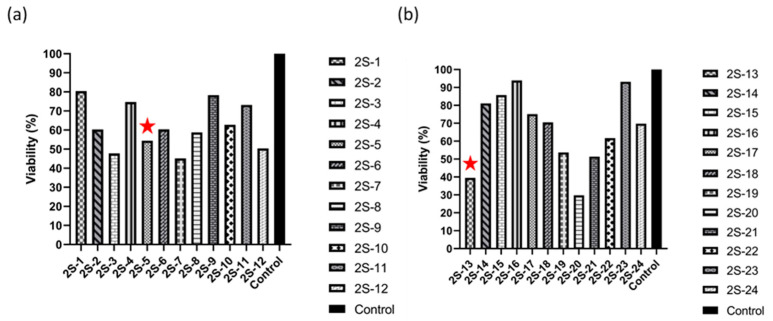
(**a**) Viability percentages of 2S-series molecules (1–12) in MDA-MB-231 cell line; (**b**) viability percentages of 2S-series molecules (13–24) in MDA-MB-231 cell line.

The cell viability results of the 2S-series compounds (2S-1 to 2S-24) in the MDA-MB-231 breast cancer cell line are shown in Figure 2. Among these, compounds 2S-5 and 2S-13 showed the most significant cytotoxic effects, reducing cell viability to below 50%. These compounds were also found to induce similar viability reduction in the 4T1 cell line (Figure 1). Based on their consistent and potent cytotoxicity across both TNBC models, 2S-5 and 2S-13 were selected for further experiments. These two compounds were derivatized with a benzamide group from the nitrogen atom of the pyridazine ring. Benzamide and substituted benzamide groups derived from the nitrogen atom are important substituents found in many commercially available drugs and are particularly preferred in inhibitors used in cancer treatment. Niraparib, veliparib, and AT533, used in cancer treatment, contain a benzamide group in their structures. Paclitaxel, a popular chemotherapy drug, has also been derivatized using a benzamide group. The amino and carbonyl groups contained in the benzamide group are important for the effectiveness of anticancer agents due to their hydrogen bonding potential [19,20,21,22].

Although several analogs (e.g., 2S-3, 2S-7, 2S-19) reduced viability to below 50% in the TNBC lines at screening concentrations, they exhibited greater cytotoxicity in non-malignant hTERT cells and therefore did not meet our pre-specified selectivity criterion (selectivity index, SI; see Methods). Consequently, these compounds were not advanced. By contrast, 2S-5 and 2S-13 satisfied both potency and selectivity requirements and were prioritized for mechanistic studies.

### 3.3. Cytotoxic Effect of 2S-5 and 2S-13 Molecules on Cell Lines

**Figure 3:**
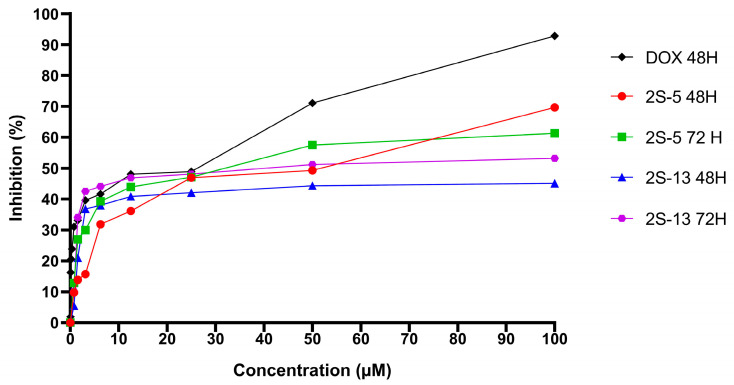
Inhibition graph of 2S-5 and 2S-13 molecules in 4T1 cell line for 48 h and 72 h. DOX in 4T1 cell line for 48 h.

The cytotoxic effects of 2S-5 and 2S-13 were evaluated on the 4T1 mouse TNBC cell line at 48 and 72 h using a range of concentrations (1.5625–100 µM). As shown in Figure 3, both compounds demonstrated a dose-dependent increase in cell inhibition. Importantly, 2S-5 exhibited a stronger cytotoxic effect compared to 2S-13 at both time points, with higher inhibition rates observed particularly at concentrations above 25 µM. These findings suggest that 2S-5 is more potent than 2S-13 in reducing 4T1 cell viability, especially at prolonged exposure times.

**Figure 4:**
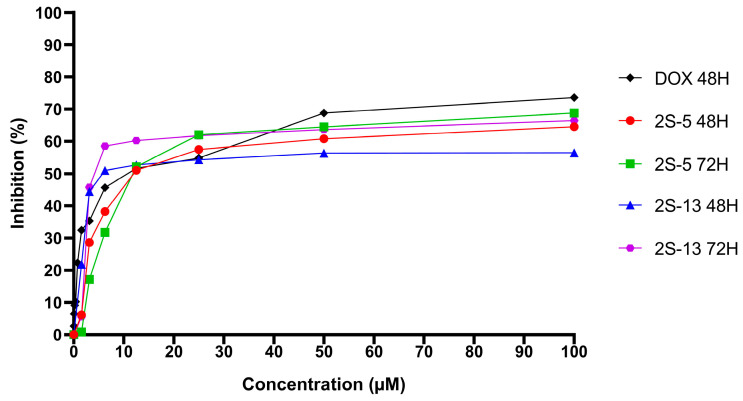
Inhibition graph of 2S-5 and 2S-13 molecules in MDA-MB-231 cell line for 48 h and 72 h. DOX in 4T1 cell line for 48 h.

The cytotoxic effects of 2S-5 and 2S-13 were further investigated in the human TNBC cell line MDA-MB-231 at 48 and 72 h with the same concentration range of 1.5625–100 µM. Both compounds exhibited a concentration- and time-dependent increase in cell inhibition, consistent with the observations in the 4T1 cell line. The 2S-5 molecules showed higher inhibition rates compared to the 2S-13 ones at both time points, particularly at higher concentrations. These results suggest that prolonged exposure and increasing concentration enhance the antiproliferative effects of both compounds in human TNBC cells, with 2S-5 showing relatively greater potency (Figure 4).

**Figure 5:**
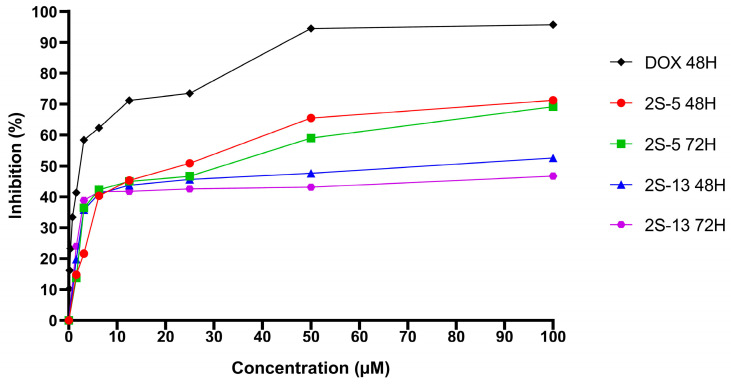
Inhibition graph of 2S-5 and 2S-13 molecules in hTERT cell line for 48 h and 72 h. DOX in 4T1 cell line for 48 h.

The cytotoxic effects of 2S-5 and 2S-13 molecules on healthy breast cells at 48 and 72 h were investigated. Increasing the concentration and incubation time for both molecules led to a decrease in inhibition values and an increase in cell viability (Figure 5).

When the anticancer effect of 2S-5 and 2S-13 molecules on all cell lines applied above and the toxicity to healthy breast cells were compared, it was determined that the 2S-13 molecule was more effective. Therefore, the mechanism of the 2S-13 molecule was selected to be examined in human and mouse TNBC breast cancer cell lines via RT-QPCR and flow cytometry experiments.

### 3.4. Cytotoxic Effect of DOX on Cell Lines

The cytotoxic effect of DOX at concentrations ranging from 1.5625 to 100 µM was examined on hTERT, 4T1, and MDA-MB-231 cell lines. The highest inhibition and cell death rate were observed in the hTERT cell line (Figure 5), followed by the MDA-MB-231 and 4T1 cell lines (Figure 3 and Figure 4, respectively).

Table 2 shows the IC_50_ and SI values of 2S-13 and DOX in the hTERT, 4T1, and MDA-MB-231 cell lines. The SI value was calculated for each substance using the following formula: SI = IC_50_ value of healthy cell/IC_50_ value of cancerous cell (4T1 and MDA-MB-231). An SI > 10 indicates that a drug is effective against tumor cells, has low toxicity against normal cells, and has high anticancer potential. When the cytotoxicity of 2S-13 was observed, it was found to have the lowest IC_50_ value in the MDA-MB-231 cell line (48H IC_50_ = 3.83 µM, 72 h IC_50_ = 2.85 µM). IC_50_ values were calculated as 48H IC_50_ = 12.4 µM and 72 h IC_50_ = 7.1 µM for the 4T1 cell line. Briefly, 2S-13 showed low cytotoxicity in the healthy breast cancer hTERT cell line, with IC_50_ values of 48H IC_50_ = 98 µM and 72 h IC_50_ = 100 µM. On the other hand, DOX showed the highest toxicity against the healthy breast cancer hTERT cell line (IC_50_ = 1.5 µM), followed by the MDA-MB-231 (IC_50_ = 6.8 µM) and 4T1 cell line (IC_50_ = 12.7 µM).

When the SI values of DOX were examined, it showed a highly toxic effect on healthy cells since the SI was <1 in the 4T1 cell line. However, the 48H SI value of the 2S-13 inhibitor was calculated to be less than 10, 7.90 µM in the 4T1 cell line. At 72 h, there was a better SI value, and this was found to be higher than 10 (SI = 14.08 µM). In the MDA-MB-231 cell line, the 2S-13 inhibitor showed a much better SI value than 4T1. While the SI value at 48 h was 25.45 µM, the SI value at 72 h was calculated to be 35.09 µM.

### 3.5. RT-qPCR Array Experiments

Changes in the gene expressions of DOX and 2S-13 inhibitors in the 4T1 mouse breast cancer cell line and MDA-MB-231 human breast cancer cell line were determined with the RT-qPCR experiment. The expression values of genes given with the 2^−ΔΔCt^ method, and, as a rule of thumb, genes with a two-fold expression increase are accepted as upregulated while genes with a decrease in expression to half the original value are accepted as downregulated.

**Figure 6:**
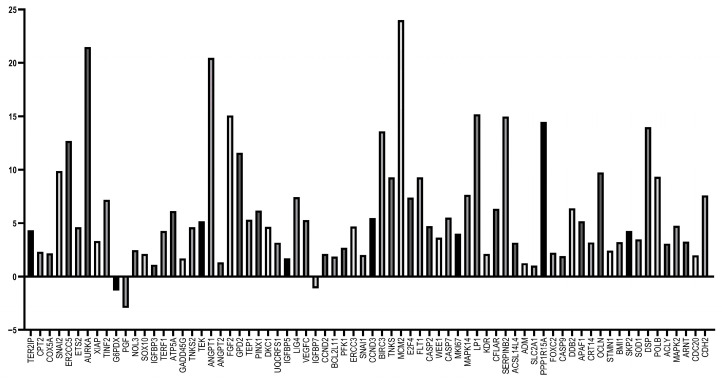
Fold change in gene expression of DOX in the 4T1 cell line.

**Figure 7:**
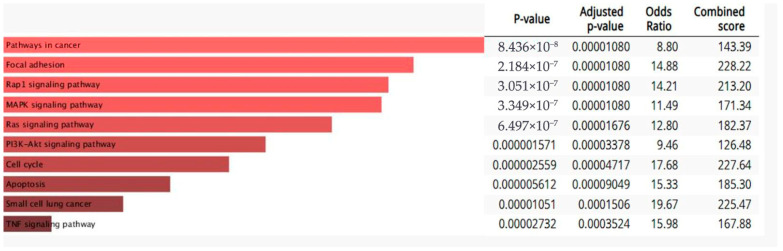
General signaling pathway analysis results of the effect of DOX on the 4T1 cell line determined by the Enrich database.

The expression alteration profiles of DOX in the 4T1 cell line are shown in Figure 6 and Figure 7. It has been observed that DOX primarily affects the cancer pathway, the repressor/activator protein 1 (RAP1) and the mitogen-activated protein kinase (MAPK) pathway, and then the RAS and phosphotidyl inositol-3-phosphate-Akt pathway.

**Figure 8:**
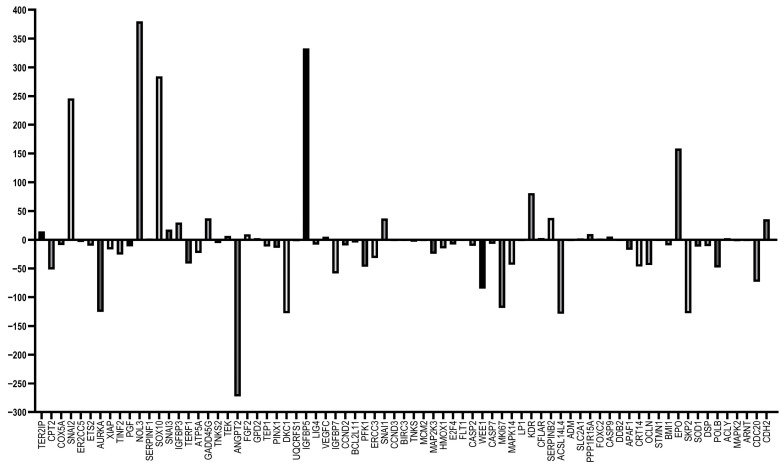
Fold change in gene expression in the 4T1 cell line of 2S-13.

**Figure 9:**
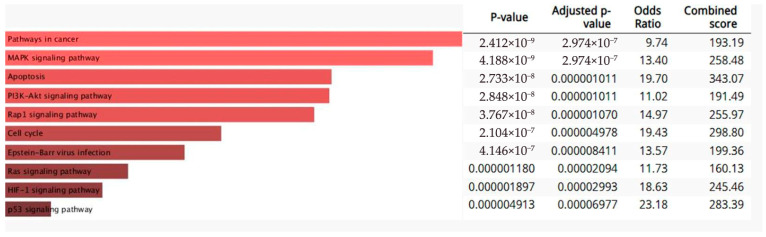
General signaling pathway analysis results of the effect of 2S-13 on the 4T1 cell line determined by the Enrich database.

Some signaling pathways that changed in terms of gene expression in the presence of 2S-13 in the 4T1 cell line are pathways in cancer, the MAPK signaling pathway, apoptosis, the PI3K-Akt signaling pathway, and then the PI3K-Akt signaling pathway, as well as the RAP1 and cell cycle pathways (Figure 8 and Figure 9).

**Figure 10:**
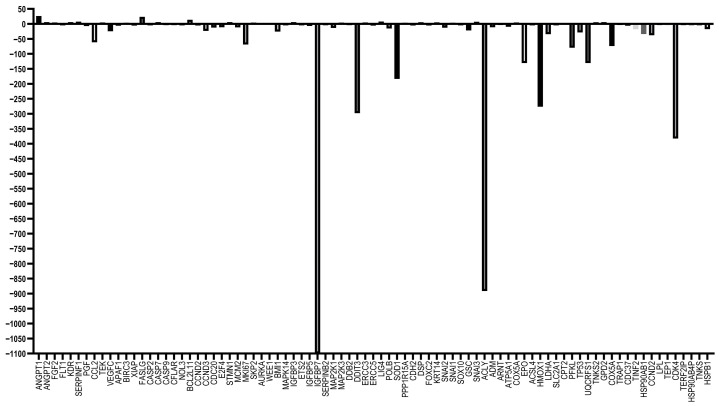
Fold change in gene expression in the MDA-MB-231 cell line of DOX.

The altered expression profile in the MDA-MB-231 cell line following treatment with DOX is shown in Figure 10. DOX caused a decrease in the expression of most genes, as shown in the following figures.

**Figure 11:**
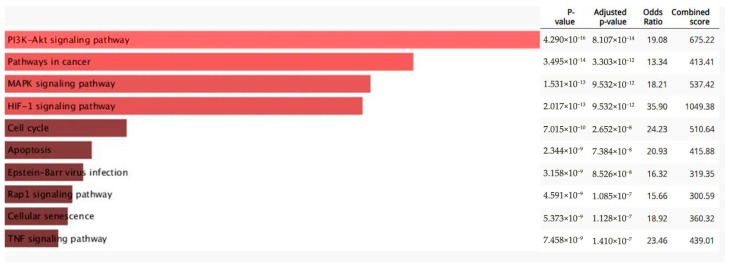
General signaling pathway analysis results in the Enrich database of the effect on the MDA-MB-231 cell line after treatment with DOX.

Key signaling pathways that changed by gene expressions in the presence of DOX in the 4T1 cell line are the PI3K-Akt signaling pathway, pathways in cancer, the MAPK signaling pathway, the hypoxia-inducible factor 1 (HIF-1) pathway, the cell cycle pathway, apoptosis and the RAP1 signaling pathway (Figure 11).

**Figure 12:**
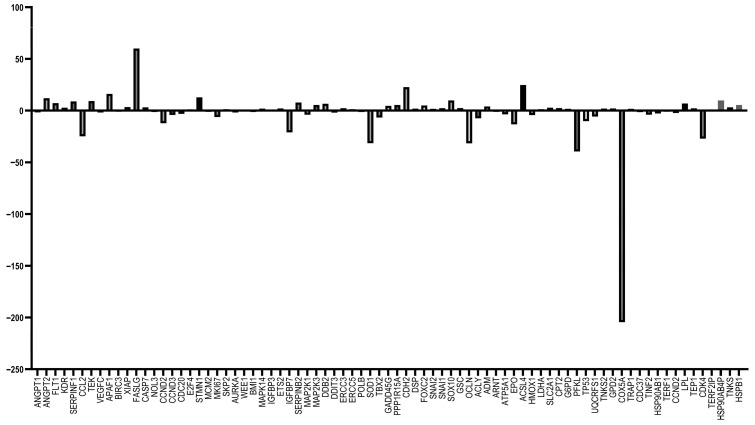
Fold change in gene expression in MDA-MB-231 cell line after treatment with 2S-13.

**Figure 13:**
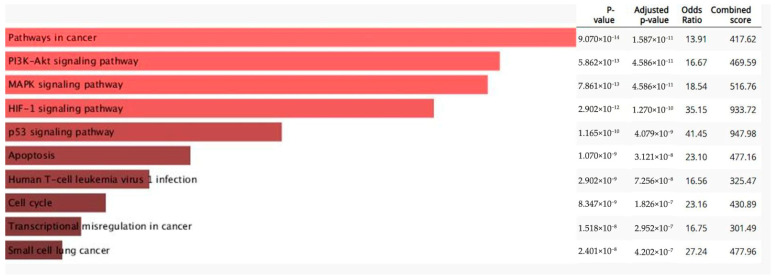
General signaling pathway analysis results in the Enrich database of the effect on the MDA-MB-231 cell line after treatment with 2S-13.

The altered expression profile in the MDA-MB-231 cell line after treatment with 2S-13 is shown in Figure 12 and Figure 13. Key signaling pathways that changed by gene expressions in the presence of 2S-13 in the MDA-MB-231 cell line are pathways in cancer, the PI3K-Akt signaling pathway, the MAPK signaling pathway, the hypoxia-inducible factor 1 (HIF-1) pathway, the p53 signaling pathway, the apoptotic pathway and the cell cycle pathway.

### 3.6. Cell Cycle Analysis with Flow Cytometry

**Figure 14:**
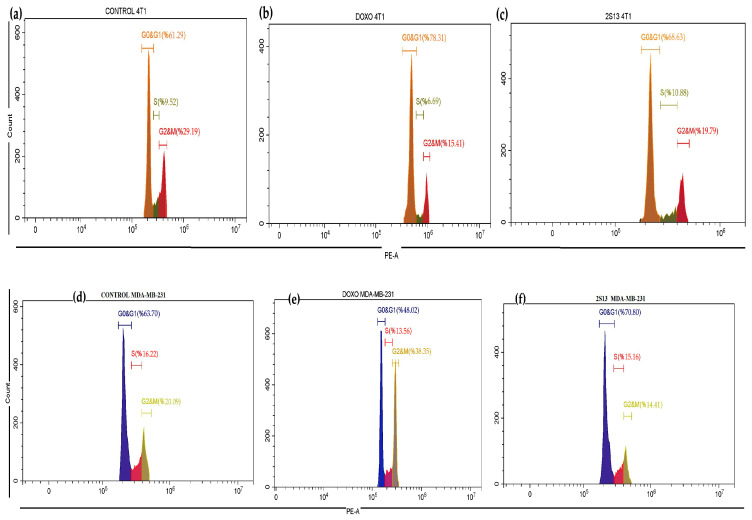
Flow cytometry and cell cycle analysis results of DOX and 2S-13 inhibitors applied to 4T1 and MDA-MB-231 cells at IC50 concentrations for 72 h. (**a**) Untreated control 4T1 cells; (**b**) DOX-treated 4T1 cells (**c**) 2S-13 treated 4T1 cells; (**d**) untreated control MDA-MB-231 cells; (**e**) DOX-treated MDA-MB-231 cells; (**f**) 2S-13-treated MDA-MB-231 cells.

Figure 14 and Table 3 show the percentages of cells in G0 & G1, S, and G2 & M phases of the results obtained from flow cytometry analysis. In 4T1 cell line, the cell population in control group was 61.29% G0 & G1, 9.52% S, and 29.19% G2 & M, while in DOX-treated cells, the proportion of cells in G0 & G1 phase increased significantly to 78.31%. On the other hand, for 4T1 cells treated with 2S-13, the cell population did not change significantly compared to that under the control conditions. In the MDA-MB-231 cell line, the percentages of cells in the control group were observed as follows: 63.70% in G0 & G1, 16.22% in S and 20.09% in the G2 & M phases; in cells treated with DOX, there was a decrease in the cell population in the G0 & G1 phase and a significant increase in the cell population in the G2 & M phase. MDA-MB-231 cells treated with 2S-13 there was a increase in the cell population in the G0 & G1 phase and a significant decrease in the cell population in the G2 & M phase.

### 3.7. Apoptosis Analysis with Flow Cytometry

This assay, used to determine the number of cells undergoing apoptosis, is based on initially staining the cells with Annexin V (AV) and propidium iodide (PI) solution, followed by detection by flow cytometry analysis. Phosphatidylserine (PS) is one of the phospholipids found in the cell membrane, and N-fluorescein isothiocyanate (FITC) was designed for the detection of apoptotic cells, which binds to PS with high affinity. In normal cells, phosphatidylserine is found in the inner membrane (the side facing the cytoplasm). However, when cells are subjected to apoptosis, phosphatidylserine is found in the outer membrane because the membrane integrity is disrupted. Red fluorescent PI dye, which binds to DNA, rapidly enters cells with damaged membranes. While living cells with intact membranes are impermeable to PI, the membranes of dead and damaged cells are permeable to PI.

**Figure 15:**
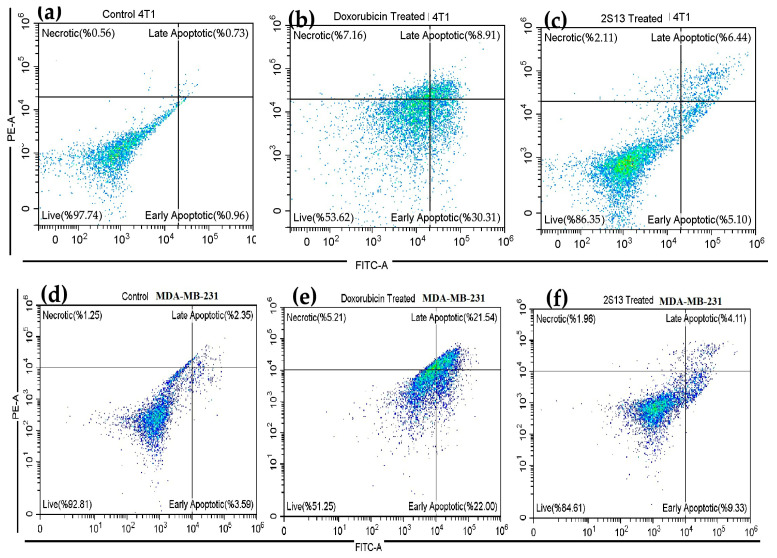
Annexin V (AV) results of DOX and 2S-13 inhibitors applied to 4T1 and MDA-MB-231 cells at IC50 concentrations for 72 h. (**a**) Control 4T1 cells without any treatment; (**b**) DOX-treated 4T1 cells; (**c**) 2S-13-applied 4T1 cells; (**d**) control MDA-MB-231 cells; (**e**) DOX-treated MDA-MB-231 cells; (**f**) MDA-MB-231 cells treated with 2S-13.

Figure 15 shows the Annexin V (AV) flow cytometry results of the DOX and 2S-13 in 4T1 and MDA-MB-231 cell lines. Q1 represents necrotic cells that are AV-negative and PI-positive. Since AV and PI are positive, they represent late apoptotic cells (Q2). Q3 indicates viable cells, as AV and PI are negative, and Q4 indicates early apoptotic cells, which are AV-positive and PI-negative.

**Figure 16:**
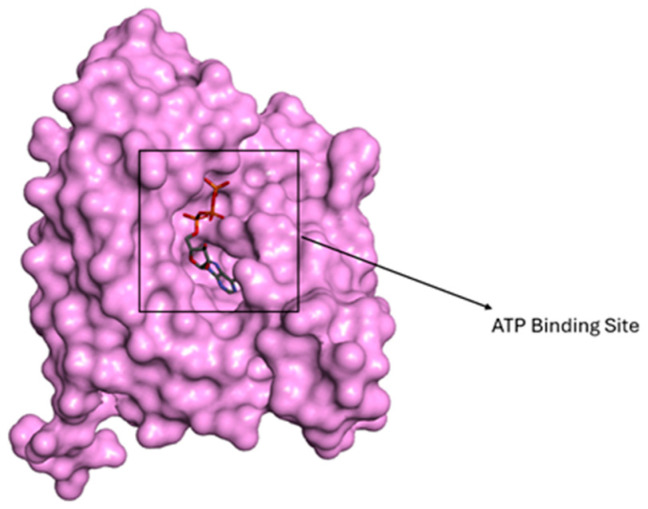
ATP and binding site in the Hsp90 NTD region.

While the percentages of 4T1 cells treated with DOX were 30.31% early apoptotic and 8.91% late apoptotic cells, the percentages of MDA-MB-231 cells treated with DOX were 22.00% early apoptotic, 21.54% late apoptotic and 5.21% necrotic cells, as can be seen in Figure 16 and Table 4.

### 3.8. Docking Studies

To understand the inhibition mechanism of Hsp90, the human Hsp90α NTD crystal structure (pdb code: 3T0Z) was examined using the molecular docking method with DOX, 2S-5 and 2S-13 inhibitors. Additionally, the interaction of ATP with Hsp90 NTD was examined. The binding energy and dissociation constant (K_D_) of inhibitors and ATP for Hsp90α NTD were calculated.

Figure 16 shows the ATP binding site in the NTD region of Hsp90. The binding energy of ATP was calculated as 7.027 kcal/mol and the dissociation constant as 7065.96 nM.

**Figure 17:**
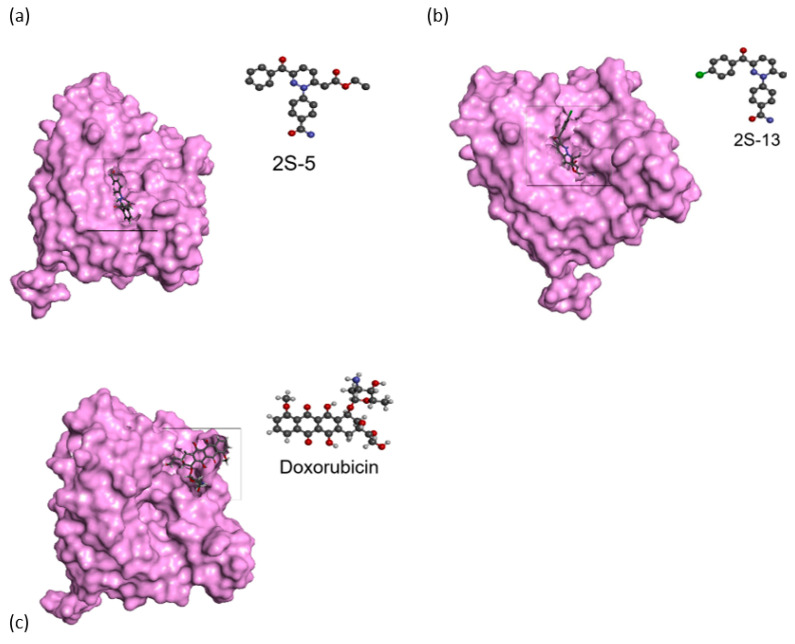
Interactions of (**a**) 2S-13; (**b**) 2S-5; (**c**) DOX with the Hsp90 NTD binding site.

When the inhibitors were examined, the binding energy of 2S-13 was 7.430 kcal/mol and that of KD was 3579.07 nM, while the binding energy of 2S-5 was 7.578 kcal/mol and the KD was 2792.66 nM; the binding energy of DOX was 7.278 kcal/mol, and the KD was calculated as 4633.6 nM. Lower binding energy values indicate stronger binding affinity between the ligand and the target protein, whereas higher values reflect weaker or non-specific interactions. The binding energies of 2S-5 and 2S-13 were higher, and these inhibitors bind to the ATP binding site of Hsp90α NTD with a higher affinity compared to ATP and DOX (Figure 17). Therefore, in the presence of these inhibitors, ATP will not be able to bind to the binding site and will not be able to carry out the ATP hydrolysis process. Thus, Hsp90 will not be able to make the appropriate conformational changes to complete the protein folding process. As a result, the chaperone function of Hsp90 will be impaired.

## 4. Discussion

In this study, 2S-series inhibitors synthesized via aryl hydrazonal compounds targeting the NTD of Hsp90 were used, and the anticancer effects of novel 2S-5 and 2S-13 compounds were investigated in MDA-MB-231, 4T1 and non-tumorigenic hTERT cell lines. The 2S-13 compound demonstrated lower IC_50_ values compared to DOX across all cell lines, with the most pronounced cytotoxicity observed in MDA-MB-231 cells. Importantly, 2S-13 exhibited limited toxicity toward hTERT cells, suggesting selective cytotoxicity.

Apoptosis studies revealed differential mechanisms triggered by 2S-13 and DOX. DOX induced a high level of early and late apoptosis (total 39.23%), while 2S-13 showed limited apoptotic activity (11.54%) in 4T1 cells. In contrast, 2S-13 effectively induced apoptosis in MDA-MB-231 cells (total 13.44%), while DOX promoted apoptosis (total 43.54%). Gene expression profiles supported these findings: DOX upregulated FASLG (23.75-fold) in MDA-MB-231, suggesting extrinsic apoptosis, whereas 2S-13 increased APAF1, CASP9, CASP7, and BCL2L11, indicating activation of the intrinsic pathway. These results underline that 2S-13 may offer a more targeted and pathway-specific mode of action in TNBC.

AURKA, an evolutionarily conserved serine/threonine kinase, regulates the initiation of mitosis, spindle fiber assembly, centrosome maturation/separation, and chromosome alignment. It is overexpressed in many human cancers, including breast, colorectal, ovarian, bladder, stomach, and pancreatic cancers. High levels of AURKA expression in approximately 73% of breast cancer patients are associated with decreased survival rates and resistance to many anticancer drugs [23]. In this study, it was shown that AURKA expression was decreased in the human TNBC breast cancer cell line MDA-MB-231treated with DOX and 2S-13, AURKA expression was increased in the 4T1 mouse TNBC breast cancer cell line treated with DOX, and AURKA expression was decreased in the 4T1 mouse TNBC breast cancer cell line treated with 2S-13. In particular, increased expression in the 4T1 TNBC cell line suggests that there will be a poor prognosis and decreased survival rate. Similarly, *MKI67*, a marker of cellular proliferation and metastasis [24], was reduced in MDA-MB-231 and 4T1 cells treated with 2S-13, indicating the inhibition of proliferation and potential for improved prognosis. However, DOX increased *MKI67* in 4T1, suggesting possible tumor aggressiveness post-treatment.

The transcription factor protein family encoding *E2F* is a gene that functions as a regulator of cell differentiation, DNA damage repair, and cell life cycle. *E2F* family genes are highly expressed in tumor tissues compared to normal tissues. It also plays a role in the regulation of telomerase expression and drug sensitivity with its tumor suppressor/oncogene-regulatory functions. *E2F4*, a member of the *E2F* family, is a strong transcriptional inhibitor and regulates differentiation and cell cycle arrest by increasing tumor growth [25]. Changes in *E2F4*, together with alterations in genes such as MCM2, WEE1, and CDC20, suggest that both DOX and 2S-13 modulate cell proliferation through E2F-related pathways. Cell cycle analysis revealed G2/M phase (38.35% with DOX) and G0/G1 phase (70.80% with 2S13) arrest in MDA-MB-231 cells post-treatment, likely due to the decreased activity of cyclins and *CDK*s, particularly *CCND2*, *CCND3*, and *CDC20*. These findings collectively imply that 2S-13 can effectively halt TNBC cell proliferation by interfering with key regulators of cell cycle progression.

*GADD45G* has critical roles in carcinogenesis processes such as cell cycle arrest, cell growth, apoptosis, DNA damage and repair [26]. Zhang et al. found that the *GADD45G* gene activates MAPK signaling by increasing the expression levels of *CSF1R*, *IGF2*, and *FGFR3*. *GADD45G* functions as a tumor suppressor in human breast cancers. Expression of *GADD45G* limits the metastasis and invasion of breast cancer cells, preventing tumor formation and breast cancer development in mice [27]. In this study, *GADD45G* expression increased in human and mouse breast cancer cell lines treated with DOX and 2S-13. It is thought that it may contribute to the activation of MAPK signaling, which is associated with cancer development.

In the MDA-MB-231 breast cancer cell, the number of cells in the G0 & G1 phase increased to 70.80% after 2S-13 application. After DOX application, the number of cells in the G2 & M phase increased to 38.35%. It is thought that the application of DOX causes a decrease in the activities of cyclins and CDKs in human TNBC breast cancer cell lines and the arrest of cancer cells in the G2 & M phase.

Markers related to cellular senescence and telomeric stability were also changed. Cellular senescence, while a tumor-suppressive process, can paradoxically promote tumor progression under certain conditions. Alterations in *TP53*, *ANGPT1*, *VEGFC*, and telomerase-related genes (*TINF2*, *TERF1*, *TEP1*, *TER2IP*, and *TNKS*) imply that 2S-13 may affect telomere dynamics and senescence in MDA-MB-231 cells, through PI3K/Akt signaling. The complexity of 2S-13’s action is highlighted by this dual role of senescence, which warrants for more research, especially in long-term in vivo settings.

Fatty acids produce lipid signaling molecules, and abnormalities in fatty acid metabolism are closely related to the growth and differentiation of cancer cells. Aggressive cancer cells are characterized by a dysregulation of fatty acid metabolism [28]. Key lipid metabolism genes, including *ACLY*, *ACSL4*, *CPT2*, and *LPL*, were downregulated by 2S-13 in MDA-MB-231 and 4T1 cells (with the exception of the latter under DOX treatment). This suggests that 2S-13 may inhibit tumor growth by limiting fatty acid biosynthesis. On the other hand, DOX treatment increased the expression of these genes in 4T1 cells, which might have led to metabolic reprogramming and a more invasive phenotype.

*HSP90AB1*, a member of the Hsp90 family, is highly expressed in most cancers and is associated with prognosis [29]. Heat shock protein 27, also called heat shock protein beta-1 (HSPB1), belongs to the small HSP family. *HSPB1* is an important molecular target that regulates many pathological processes in cancer, including drug resistance, apoptosis and metastasis [29]. This study showed that *HSP90* and *HSPB1* were significantly affected by treatment. HSP90AB1 expression was upregulated in MDA-MB-231 cells treated with 2S-13, accompanied by increased angiogenesis-related genes (*KDR*, *ANGPT2*, *TEK*). Furthermore, high HSPB1 expression may have triggered EMT-related genes (SNAI1, SNAI2, SOX10), suggesting a complex dual effect of 2S-13 on tumor plasticity. Despite this, the overall data indicate a therapeutic advantage of 2S-13, especially in the MDA-MB-231 cell lines.

In conclusion, these findings indicate that 2S-13 appears to be a promising therapeutic candidate for TNBC, through the targeted induction of apoptosis, multi-pathway regulation involving cell cycle arrest, the inhibition of fatty acid metabolism, and HSP modulation. These findings support further preclinical evaluation and open up new avenues for developing Hsp90-targeted therapies in breast cancer.

## 5. Conclusions

The results indicated that the apoptotic effect of the 2S-13 inhibitor was higher in the MDA-MB-231 cell line, and that it can prevent the growth of MDA-MB-231 cancer cells by inducing apoptosis. The 2S-13 inhibitor was found to induce cell accumulation in the G0/G1 phase in the MDA-MB-231 cell line treatment. When the expressions of genes in cancer pathways were evaluated, significant changes were found in terms of PI3K/AKT, MAPK, apoptosis, cell proliferation, cell cycle, aging, and cancer metabolism. Molecular docking studies showed that the 2S-13 inhibitor has higher binding energy. It can bind to the ATP binding site of Hsp90 NTD with higher affinity compared to ATP and DOX. Thus, it may be inferred from the results that 2S-13 has a greater inhibitory effect on Hsp90. All these results showed that 2S-13 is a promising drug candidate in the MDA-MB-231 cell line.

## Figures and Tables

**Table 1:** Some data on the synthesized pyridazine derivative compounds.

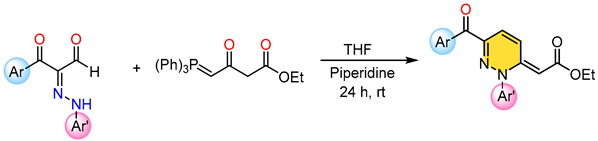					
Compound	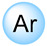	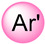	Melting Point (°C) Yield	Molecular Formula	HRMS Calculated (Found)
2S-1	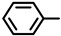	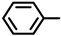	182–183 %74	C_21_H_18_N_2_O_3_	(M+H): 347.1390 (347.1393)
2S-2	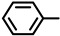	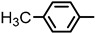	149–150 %76	C_22_H_20_N_2_O_3_	(M+H): 361.1547 (361.1548)
2S-3	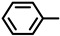	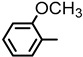	184–185 %78	C_22_H_20_N_2_O_4_	(M+H): 377.1496 (377.1507)
2S-4	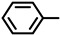	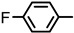	193–194 %68	C_21_H_17_FN_2_O_3_	(M+H): 365.1296 (365.1301)
2S-5	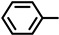	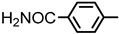	222–223 %72	C_22_H_19_N_3_O_4_	(M+H): 390.1448 (390.1452)
2S-6	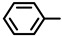	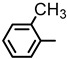	190–191 %63	C_22_H_20_N_2_O_3_	(M+H): 361.1547 (361.1544)
2S-7	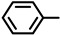	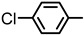	155–156 %69	C_21_H_17_ClN_2_O_3_	(M+H): 381.1006 (381.1001)
2S-8	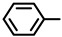	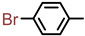	191–192 %71	C_21_H_17_BrN_2_O_3_	(M-): 424.0428 (424.0391)
2S-9	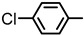	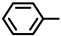	219–220 %79	C_21_H_17_ClN_2_O_3_	(M+H): 381.1000 (381.1006)
2S-10	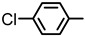	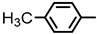	305–306 %71	C_22_H_19_ClN_2_O_3_	(M+H): 395.1157 (395.1154)
2S-11	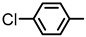	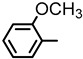	205–206 %71	C_22_H_19_ClN_2_O_4_	(M+H): 411.1106 (411.1113)
2S-12	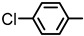	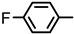	193–194 %68	C_21_H_16_ClFN_2_O_3_	(M+H): 399.0906 (399.0906)
2S-13	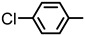	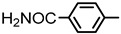	310–311 %70	C_22_H_18_ClN_3_O_4_	(M+H): 424.1059 (424.1056)
2S-14	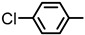	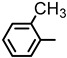	199–200 %68	C_22_H_19_ClN_2_O_3_	(M+H): 395.1157 (395.1159)
2S-15	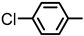	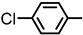	206–207 %77	C_21_H_16_Cl_2_N_2_O_3_	(M+H): 415.0611 (415.0611)
2S-16	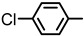	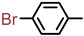	172–173 %69	C_21_H_16_BrClN_2_O_3_	(M+H): 459.0111 (-)
2S-17	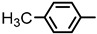	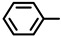	193–194 %69	C_22_H_20_N_2_O_3_	(M+H): 361.1547 (361.1548)
2S-18	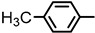	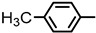	178–179 %73	C_23_H_22_N_2_O_3_	(M+H): 375.1703 (375.1709)
2S-19	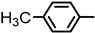	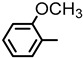	171–172 %72	C_23_H_22_N_2_O_4_	(M+H): 391.1613 (391.1648)
2S-20	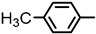	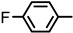	191–192 %75	C_22_H_19_FN_2_O_3_	(M+H): 379.1452 (379.1449)
2S-21	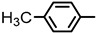	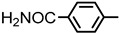	237–238 %73	C_23_H_21_N_3_O_4_	(M+H): 404.1605 (404.1606)
2S-22	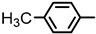	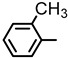	166–167 %64	C_23_H_22_N_2_O_3_	(M+H): 375.1703 (375.1709)
2S-23	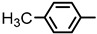	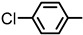	174–175 %76	C_22_H_19_ClN_2_O_3_	(M+H): 395.1157 (395.1163)
2S-24	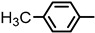	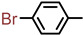	193–194 %72	C_22_H_19_BrN_2_O_3_	(M+H): 439.0652 (439.0652)

**Table 2:** IC_50_ and SI values of 2S-13 and DOX inhibitors.

Cell Lines	Inhibitors (IC_50_ (µM)) 48 h		Inhibitors (IC_50_ (µM)) 72 h	Selectivity Index (SI) 48 h		Selectivity Index (SI) 72 h
	2S-13	DOX	2S-13	2S-13	DOX	2S-13
hTERT	98	1.5	100			
4T1	12.4	12.7	7.1	7.90	0.118	14.08
MDA-MB-231	3.83	6.8	2.85	25.45	0.220	35.09

**Table 3:** Percentage of cells in G0 & G1, S and G2 & M phases of the cell cycle obtained from flow cytometry analysis.

	G0 & G1	S	G2 & M
4T1-CONTROL	% 61.29	% 9.52	% 29.19
MDA-MB-231-CONTROL	% 63.70	% 16.22	% 20.19
4T1-2S-13	% 68.63	% 10.88	% 19.79
MDA-MB-231-2S-13	% 70.80	% 15.16	% 14.41
4T1-DOX	% 78.31	% 6.69	% 15.41
MDA-MB-231-DOX	% 48.02	% 13.56	% 38.35

**Table 4:** Cell population in live, early apoptotic, late apoptotic, and necrotic stages after treatment with DOX and 2S-13 in 4T1 and MDA-MB-231 cells.

	Live	Early Apoptotic	Late Apoptotic	Necrotic
4T1-CONTROL	97.74%	0.96%	0.73%	0.56%
MDA-MB-231-CONTROL	92.81%	3.59%	2.35%	1.25%
4T1-DOX	53.62%	30.31%	8.91%	7.16%
MDA-MB-231-DOX	51.25%	22.00%	21.54%	5.21%
4T1-2S-13	86.35%	5.10%	6.44%	2.11%
MDA-MB-231-2S-13	84.61%	9.33%	4.11%	1.96%

## Data Availability

The original contributions presented in this study are included in the article. Further inquiries can be directed to the corresponding author.

## Abbreviations

The following abbreviations are used in this manuscript:

HSP	Heat shock protein
TNBC	Triple-negative breast cancer
DOX	Doxorubicin
ER+	Estrogen receptor
PR+	Progesterone receptor
HER2+	Human epidermal receptor 2-expressing breast cancer
TOP2	Topoisomerase II
DSBs	Double-strand DNA breaks
NTD	Amino terminal part of Hsp
SI	Selectivity index
MTT	3-(4,5-Dimethylthiazol-2-yl)-2,5-Diphenyltetrazolium Bromide
MAPK	Mitogen-activated protein kinase
PI3K	Phosphatidylinositol-3-kinase
AKT	Protein kinase B
